# Safety, pharmacokinetics and efficacy of selumetinib in Chinese adult and paediatric patients with neurofibromatosis type 1 and inoperable plexiform neurofibromas: The primary analysis of a phase 1 open‐label study

**DOI:** 10.1002/ctm2.1589

**Published:** 2024-03-08

**Authors:** Zhichao Wang, Xin Zhang, Chunyan Li, Yangbo Liu, Xiaoyun Ge, Jiajia Zhao, Xiaojun Yuan, Qingfeng Li

**Affiliations:** ^1^ Shanghai Ninth People's Hospital Affiliated to Shanghai JiaoTong University School of Medicine Shanghai China; ^2^ Xinhua Hospital Affiliated to Shanghai JiaoTong University School of Medicine Shanghai China; ^3^ AstraZeneca Global R&D (China) Co. Ltd. Shanghai China

Dear Editor,

We present data from a phase 1 study (NCT04590235; D1346C00011; CTR20200357) investigating the safety and pharmacokinetics (PK) of selumetinib for the first time in Chinese paediatric and adult patients with neurofibromatosis type 1 (NF1) and inoperable plexiform neurofibromas (PN).

In NF1, neurofibromin is dysfunctional, causing constitutive activation of the rat sarcoma (RAS)–rapidly accelerated fibrosarcoma (RAF)–mitogen‐activated protein kinase kinase (MEK)–extracellular signal‐regulated kinase (ERK) pathway.^1^ Patients with NF1 can develop PN that can cause pain, disfigurement, functional limitations, neurologic deficits and internal organ compression.^2^ Although surgery is a treatment option for PN, the risks often outweigh the benefits.^2^

Selumetinib (ARRY‐142886, AZD6244), an oral MEK1/2 inhibitor, stops NF1–PN growth by blocking RAS signalling.^3^, ^4^ Based on results of the pivotal SPRINT trial,^4^ selumetinib was approved in the USA (aged ≥2 years), European Union (aged ≥3 years) and China (aged ≥3 years) for paediatric patients with NF1 and symptomatic, inoperable PN.^5^, ^6^, ^7^ In SPRINT, patients receiving selumetinib reported improvements in quality of life during treatment and durable PN shrinkage.^4^ However, there was an unmet need for clinical trials designed to evaluate the safety, PK and efficacy of selumetinib in Chinese patients with NF1–PN.

This phase 1, single‐arm study, conducted at two centres in China, evaluated selumetinib capsules at a dosage of 25 mg/m^2^ twice daily in two independent cohorts (adult and paediatric; Figure 1). We present data from the primary analysis, which was required for selumetinib registration in China, and was performed after the last dosed patient had completed their cycle 10 visit (data cut‐off [DCO] 16 August 2022). At primary DCO, patients were expected to have completed two post‐baseline response assessments (at cycles 4 and 8).

**FIGURE 1:**
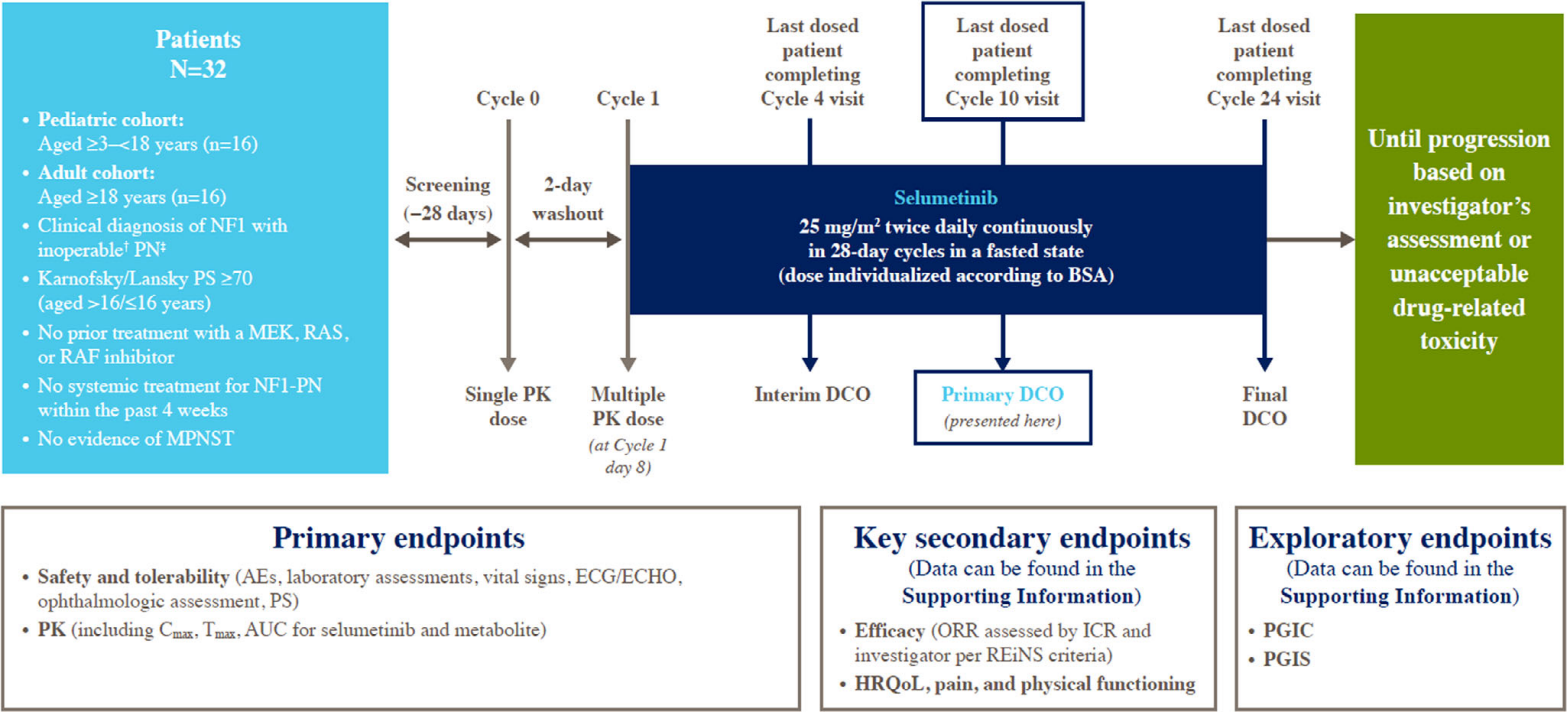
Study design. ^†^Inoperable PN were defined as PN that cannot be completely removed by surgery without risk of substantial morbidity due to encasement of, or close proximity to, vital structures, invasiveness, or high vascularity of the PN. ^‡^Patients must have had ≥1 measurable PN (≥3 cm). The target PN (refer to Supporting Information for further information) was defined as the most clinically relevant PN; only one non‐target PN could be selected, if any. Intensive PK samples were collected after single and multiple dosing. The SRC evaluated preliminary tolerability and safety data, and PK data (if available) after the first six patients in both cohorts had approximately three cycles of treatment. Additional enrollment was initiated per SRC recommendation. Long‐term post‐treatment safety follow‐up assessments will be conducted for 1 year (at 6‐ and 12‐month post‐treatment) for paediatric patients only. Abbreviations: AE, adverse event; AUC, area under the concentration–time curve; BSA, body surface area; *C*_max_, maximum plasma concentration; DCO, data cut‐off; ECG, electrocardiogram; ECHO, echocardiogram; HRQoL, health‐related quality of life; ICR, independent central review; MEK, mitogen‐activated protein kinase kinase; MPNST, malignant peripheral nerve sheath tumour; NF1, neurofibromatosis type 1; ORR, objective response rate; PGIC, patient's global impression of change; PGIS, patient's global impression of severity; PK, pharmacokinetics; PN, plexiform neurofibroma; PS, performance status; RAF, rapidly accelerated fibrosarcoma; RAS, rat sarcoma; REiNS, response evaluation in neurofibromatosis and schwannomatosis; SRC, Safety Review Committee; *T*_max_, time to reach maximum plasma concentration.

Overall, 16 adult and 16 paediatric patients received selumetinib (Table 1). One adult discontinued treatment due to the patient's decision but remained on the study; all paediatric patients continued to receive treatment at primary DCO. The reported safety profile was consistent with the known safety profile of selumetinib.^5^, ^6^ All patients in the study experienced adverse events (AEs); most experienced treatment‐related AEs (Table 2). The most common AEs in the adult and paediatric cohorts were dermatitis acneiform (*n *= 13; 81%) and pyrexia (*n *= 6; 38%), respectively; all were grade 1/2 events and were reported in SPRINT.^4^, ^5^ The most common treatment‐related AEs were dermatitis acneiform (*n *= 13; 81%) in adult patients, and decreased blood albumin and paronychia in paediatric patients (both, *n *= 4; 25%). One (6%) adult patient reported a serious adverse event (SAE; grade 3 pulmonary tuberculosis, not treatment related) leading to dose interruption. One (6%) adult patient experienced an AE leading to dose reduction and two (13%) experienced AEs leading to dose interruption (Table 2). One (6%) paediatric patient reported SAEs (two grade 3 sepsis and one grade 3 urinary tract infection, neither were treatment related). Three (19%) paediatric patients experienced AEs leading to dose interruption; none experienced AEs leading to dose reduction (Table 2). Two (13%) adult patients and one (6%) paediatric patient reported grade ≥3 AEs; similar to SPRINT phase 2 Stratum 1, most AEs, such as increased blood creatine phosphokinase and paronychia, were grade 1/2.^4^ No grade 4 AEs were reported at primary DCO; no AEs led to death or selumetinib discontinuation. No unexpected clinically significant trends of change were observed for laboratory assessments, vital signs or electrocardiogram/echocardiogram in either cohort. No abnormal reports of bone growth or Tanner stages were reported in the paediatric cohort.

**TABLE 1:** Baseline demographics and clinical characteristics.

Characteristic	Adult patients (*n *= 16)	Paediatric patients (*n *= 16)
Sex, *n* (%)		
Male	9 (56)	9 (56)
Female	7 (44)	7 (44)
Age (years), median (range)	24.5 (18−51)	11.0 (4−16)
Time from NF1 diagnosis^a^ (years), median (range)	4.1 (.1−34.5)	2.7 (.4−14.3)
NF1 diagnostic criteria, *n* (%)		
Any café‐au‐lait macules	16 (100)	16 (100)
≥6 café‐au‐lait macules	16 (100)	16 (100)
Freckling in axilla or groin	15 (94)	16 (100)
≥2 Lisch nodules	12 (75)	10 (63)
A first‐degree relative with NF1	8 (50)	1 (6)
A distinctive bony lesion	4 (25)	0
Optic glioma	0	0
Target PN location, *n* (%)		
Extremity	6 (38)	5 (31)
Trunk	5 (31)	3 (19)
Head and neck	1 (6)	2 (13)
Neck or trunk	0	1 (6)
Trunk or extremity	0	1 (6)
Head	4 (25)	1 (6)
Other	0	3 (19)
Any target PN‐related morbidity^b^, *n* (%)		
Pain	4 (25)	14 (88)
Disfigurement	2 (13)	5 (31)
Motor	4 (25)	4 (25)^c^
Airway obstruction	0	1 (6)
Bowel or bladder dysfunction	0	1 (6)
Vision loss	2 (13)	0
Other^d^	4 (25)	16 (100)
Target PN volume (mL), median (range)	691.7 (46.2−7746.3)	517.4 (47.6−2664.4)
≥1 prior NF1‐ or PN‐related treatment, *n* (%)	13 (81)	12 (75)
Medical treatment	0	3 (19)
Surgery	13 (81)	10 (63)

Abbreviations: NF1, neurofibromatosis type 1; PN, plexiform neurofibroma.

^a^ To start of selumetinib treatment.

^b^ Patients could have more than one target PN‐related morbidity documented.

^c^ All instances were reduced range of motion.

^d^ Details of other target PN‐related morbidities are not available.

**TABLE 2:** Adverse event (AE) profile in adult and paediatric patients at primary data cut‐off (16 August 2022; after the last dosed patient completed cycle 10, day 28).

Patients experiencing AEs, *n* (%)	All	Treatment related
Adult patients (*n* = 16)		
Any AE (any grade)	16 (100)	16 (100)
Any AE grade ≥3	2 (13)^a^	1 (6)
Any AE grade 5	0	0
Any SAE	1 (6)	0
AEs leading to dose modifications^b^	3 (19)^c^	2 (13)^d^
AEs occurring in ≥10% of patients		
Dermatitis acneiform	13 (81)	13 (81)
Aspartate aminotransferase increased	10 (63)	10 (63)
Hyperphosphataemia	9 (56)	9 (56)
Conjunctivitis	7 (44)	4 (25)
Blood creatine phosphokinase increased	5 (31)	5 (31)
Hyperuricaemia	5 (31)	5 (31)
Alanine aminotransferase increased	4 (25)	4 (25)
Blood alkaline phosphatase increased	4 (25)	4 (25)
Blood lactate dehydrogenase increased	4 (25)	4 (25)
Paronychia	4 (25)	4 (25)
Trichiasis	3 (19)	1 (6)
Anaemia	2 (13)	2 (13)
Diarrhoea	2 (13)	2 (13)
Dry mouth	2 (13)	2 (13)
Dry skin	2 (13)	2 (13)
Skin ulcer	2 (13)	2 (13)
Urinary tract infection	2 (13)	2 (13)
Paediatric patients (*n *= 16)		
Any AE (any grade)	16 (100)	14 (88)
Any AE grade ≥3	1 (6)^e^	0
Any AE grade 5	0	0
Any SAE	1 (6)	0
AEs leading to dose modifications^b^	3 (19)^f^	2 (13)^g^
AEs occurring in ≥10% of patients		
Pyrexia	6 (38)	1 (6)
Blood albumin decreased	5 (31)	4 (25)
Alanine aminotransferase increased	4 (25)	2 (13)
Aspartate aminotransferase increased	4 (25)	2 (13)
Paronychia	4 (25)	4 (25)
Blood creatine phosphokinase increased	3 (19)	2 (13)
Eczema	3 (19)	2 (13)
Hyperuricaemia	3 (19)	1 (6)
Rash	3 (19)	3 (19)
Upper abdominal pain	2 (13)	0
Cough	2 (13)	0
Dry skin	2 (13)	1 (6)
Haemoglobin decreased	2 (13)	2 (13)
Ocular hypertension	2 (13)	2 (13)
Rhinorrhoea	2 (13)	0
Stomatitis	2 (13)	1 (6)
Upper respiratory tract infection	2 (13)	0
Vomiting	2 (13)	0

Abbreviations: D‐j, double j; SAE, serious adverse event.

^a^ Grade 3 paronychia considered to be treatment related and grade 3 pulmonary tuberculosis not considered to be treatment related.

^b^ Interruption or reduction.

^c^ Grade 3 paronychia (treatment related; dose interruption and subsequent dose reduction), grade 2 haematoma (treatment related; dose interruption) and grade 3 pulmonary tuberculosis (not treatment related; dose interruption).

^d^ Grade 3 paronychia (treatment related; dose interruption and subsequent dose reduction) and grade 2 haematoma (treatment related; dose interruption).

^e^ Two instances of grade 3 sepsis and one instance of grade 3 urinary tract infection (possibly due to D‐j urethral catheterisation) that led to dose interruption; these were not considered to be treatment related.

^f^ Grade 3 sepsis (not treatment related; dose interruption), grade 2 ocular hypertension (treatment related; dose reduction) and grade 1 rash (treatment related; dose interruption).

^g^ Grade 2 ocular hypertension (treatment related; dose reduction) and grade 1 rash (treatment related; dose interruption).

Selumetinib was rapidly absorbed in both cohorts; median (range) time to reach maximum plasma concentration (*T*_max_) at steady state was 1.5 h (.5–1.6 h) and 1.5 h (.5–3.0 h) in the adult and paediatric cohorts, respectively (Table 3). In adults, the median (range) *T*_max_ for a single dose (1.0 h [.5–1.5 h]) and at steady state (1.5 h [.5−1.6 h]; Table 3) observed in this study showed similarity with those published for White (1.0 h [1.0−4.0 h]) and Asian (1.0 h [1.0−4.0 h]) patients in a pooled analysis of healthy subjects.^8^ In adult and paediatric cohorts, the metabolite‐to‐parent ratios of area under the concentration–time curve (AUC) from time 0−12 h at steady state (AUC_0−12 h,ss_) and maximum plasma concentration (*C*_max_) at steady state (*C*_max,ss_) were ≤.074 and ≤.085, respectively, indicating a higher exposure to selumetinib than its active metabolite, N‐desmethyl selumetinib. The single‐dose *C*_max_ reported in the paediatric and adult cohorts were 871 and 1285 ng/mL, respectively (Table 3). A rapid elimination profile was observed in both cohorts; selumetinib had a mean half‐life of 7.49 and 7.20 h in adult and paediatric patients, respectively. There was no obvious accumulation after multiple dosing. In the adult cohort, AUC_0−12 h,ss_ and *C*_max,ss_ accumulation ratios were 1.34 and .96, respectively. In the paediatric cohort, AUC_0−12 h,ss_ and *C*_max,ss_ accumulation ratios were 1.51 and 1.29, respectively. Selumetinib had a systemic exposure temporal change parameter of AUC ≤1.13 and ≤1.26 in the adult and paediatric cohorts, respectively (Table 3). Low‐to‐moderate variability of PK exposure was observed in adult patients at steady state with a geometric coefficient of variation (gCV) of 20%−32% for AUC_0−12,ss_ and *C*_max,ss_ for selumetinib and its metabolite. In paediatric patients, moderate variability of PK exposure was observed at steady state with a gCV ranging from 32% to 44% for AUC_0−12 h,ss_ and *C*_max,ss_ for selumetinib and its metabolite (Tables 3 and S1). The observed selumetinib PK profile was similar to that seen in SPRINT^9^ and in a phase 1 Japanese trial.^10^ PK parameters of N‐desmethyl selumetinib are in Table S1. The geometric mean plasma concentration–time profiles are shown in Figure S1.

**TABLE 3:** Selumetinib pharmacokinetics (PK) parameters following single and multiple doses.

	Single dose		Steady state	
PK parameter	Adult (*n *= 16)	Paediatric (*n *= 16)	Adult (*n *= 16)	Paediatric (*n *= 16)
*T*_max_ (h), median (range)	1.0 (.5−1.5)	1.5 (1.0−3.0)	1.5 (.5−1.6)	1.5 (.5−3.0)
*C*_max_ (ng/mL), GeoMean (gCV%)	1285 (23.90%)	871 (68.33%)	1168 (32.19%)	1032 (41.28%)
AUC_0−12 h_ (ng h/mL), GeoMean (gCV%)	2929 (22.73%)	2015 (49.97%)	3932 (19.56%)	2961 (43.82%)
AUC_last_ (ng h), GeoMean (gCV%)	3370 (22.60%)	2343 (47.56%)	–	–
AUC_inf_ (ng h/mL), GeoMean (gCV%)	3479 (23.28%) (*n *= 15)	2415 (47.36%)	–	–
*t*_1/2_ *λ*_z_ (h), AriMean ± SD	7.490 ± 2.502 (*n *= 15)	7.200 ± 1.537	–	–
*R*_ac_ AUC_0−12 h_, AriMean ± SD	–	–	1.339 ± .241	1.511 ± .377
*R*_ac_ *C*_max_, AriMean ± SD	–	–	.9596 ± .3490	1.289 ± .551
TCP, AriMean ± SD	–	–	1.128 ± .217 (*n *= 14)	1.262 ± .329

Abbreviations: AriMean, arithmetic mean; AUC_0−12 h_, area under the concentration–time curve from time 0−12 h; AUC_inf_, area under the concentration–time curve from zero to infinity; AUC_last_, area under the concentration–time curve from zero to the last measurable concentration; *C*_max_, maximum plasma concentration; gCV, geometric coefficient of variation; GeoMean, geometric mean; *R*_ac_ AUC_0−12 h_, accumulation ratio based on AUC_0−12 h_; *R*_ac_ *C*_max_, accumulation ratio based on *C*_max_; SD, standard deviation; *t*_1/2_ *λ*_z_, terminal elimination of half‐life; TCP, temporal change parameter in systemic exposure; *T*_max_, time to reach maximum plasma concentration.

Selumetinib showed promising efficacy and improvements from baseline in health‐related quality of life in both cohorts (Supporting Information). Efficacy was a secondary endpoint. Therefore, these first published adult efficacy data should be interpreted with caution and considered preliminary. Efficacy and safety of selumetinib in adults with NF1–PN are being assessed in an ongoing randomised, double‐blind, placebo‐controlled, two‐arm, global phase III study (KOMET; NCT04924608; 146 participants).

Overall, within context of limitations (single‐arm trial, small sample size), this study demonstrated that selumetinib has an acceptable benefit–risk profile. Therefore, selumetinib may address the unmet medical need for patients with NF1–PN in China.

## AUTHOR CONTRIBUTIONS

*Conceptualisation*: Zhichao Wang, Xin Zhang, Qingfeng Li, Chunyan Li, Yangbo Liu, Xiaoyun Ge, Jiajia Zhao and Xiaojun Yuan. *Resources*: Zhichao Wang, Xin Zhang, Qingfeng Li, Xiaoyun Ge and Xiaojun Yuan. *Data curation*: Zhichao Wang, Xin Zhang, Qingfeng Li, Chunyan Li, Xiaoyun Ge, Jiajia Zhao and Xiaojun Yuan. *Software*: Zhichao Wang, Xin Zhang, Qingfeng Li and Xiaojun Yuan. *Formal analysis*: Zhichao Wang, Xin Zhang, Qingfeng Li, Chunyan Li, Yangbo Liu, Xiaoyun Ge, Jiajia Zhao and Xiaojun Yuan. *Supervision*: Zhichao Wang, Xin Zhang, Qingfeng Li, Chunyan Li, Yangbo Liu, Xiaoyun Ge, Jiajia Zhao and Xiaojun Yuan. *Funding acquisition*: Zhichao Wang and Qingfeng Li. *Validation*: Zhichao Wang, Xin Zhang, Qingfeng Li, Chunyan Li, Yangbo Liu, Xiaoyun Ge, Jiajia Zhao and Xiaojun Yuan. *Investigation*: Zhichao Wang, Xin Zhang, Qingfeng Li, Chunyan Li, Xiaoyun Ge, Jiajia Zhao and Xiaojun Yuan. *Visualisation*: Zhichao Wang, Xin Zhang, Qingfeng Li, Chunyan Li, Xiaoyun Ge, Jiajia Zhao and Xiaojun Yuan. *Methodology*: Zhichao Wang, Xin Zhang, Qingfeng Li, Yangbo Liu, Jiajia Zhao and Xiaojun Yuan. *Writing—original draft*: Zhichao Wang, Xin Zhang, Qingfeng Li and Xiaojun Yuan. *Project administration*: Zhichao Wang, Xin Zhang, Qingfeng Li, Chunyan Li, Xiaoyun Ge, Jiajia Zhao and Xiaojun Yuan. *Writing—review and editing*: Zhichao Wang, Xin Zhang, Qingfeng Li, Chunyan Li, Xiaoyun Ge, Jiajia Zhao and Xiaojun Yuan.

## CONFLICT OF INTEREST STATEMENT

Z.W. and Q.L. received research grants from the National Natural Science Foundation of China and the Science and Technology Commission of Shanghai Municipality. Z.W., X.Z., C.L., X.G., Y.L., J.Z., X.Y. and Q.L. received support for the clinical study from AstraZeneca as part of an alliance between AstraZeneca and Merck Sharp & Dohme LLC, a subsidiary of Merck & Co., Inc., Rahway, NJ, USA, editorial/medical writing assistance from Connie Feyerherm and Emily Clark of OPEN Health Communications (London, UK), and financial support from Alexion, AstraZeneca Rare Disease. J.Z., Y.L., X.G. and C.L. report employment at AstraZeneca.

## ETHICS STATEMENT

The clinical study protocol and participant informed consent documents were submitted to the Ethics Committee for review and were approved before the initiation of the study (paediatric, XHEC‐A‐2020‐019‐1; adult, SH9H‐2020‐C9‐3). All the participants provided written informed consent.

## CONSENT FOR PUBLICATION

All authors declare that they agree to publish this present manuscript with no conflicts of interest.

## Supporting information

Supporting information

## Data Availability

Alexion will consider requests for disclosure of clinical study participant‐level data, provided that participant privacy is assured through methods such as data de‐identification, pseudonymisation or anonymisation (as required by applicable law), and if such disclosure was included in the relevant study informed consent form or similar documentation. Qualified academic investigators may request participant‐level clinical data and supporting documents (statistical analysis plan and protocol) pertaining to Alexion‐sponsored studies. Further details regarding data availability and instructions for requesting information are available in the Alexion Clinical Trials Disclosure and Transparency Policy, available at https://alexion.com/our‐research/research‐and‐development. Link to data request form: https://alexion.com/contact‐alexion/medical‐information.
