# Supplementary material for: Safety, pharmacokinetics and efficacy of selumetinib in Chinese adult and paediatric patients with neurofibromatosis type 1 and inoperable plexiform neurofibromas: The primary analysis of a phase 1 open‐label study
Source: Clin Transl Med. 2024 Mar 8;14(3):e1589. doi: 10.1002/ctm2.1589 (PMC10921232; doi:10.1002/ctm2.1589)
Supplement: Supplementary file 1 — Supporting information [file CTM2-14-e1589-s001.docx]

**Informative title**

**Safety, pharmacokinetics, and efficacy of selumetinib in Chinese adult and pediatric patients with neurofibromatosis type 1 and inoperable plexiform neurofibromas: the primary analysis of a Phase 1 open-label study**

**Running title**

**Selumetinib for inoperable NF1-PN in China**

**Authors**

Zhichao Wang,^1#^ Xin Zhang,^2#^ Chunyan Li,^3^ Yangbo Liu,^3*^ Xiaoyun Ge,^3^ Jiajia Zhao,^3^ Xiaojun Yuan,^2^ Qingfeng Li^1^

**Affiliations**

*^1^Shanghai Ninth People's Hospital affiliated to Shanghai JiaoTong University School of Medicine, Shanghai, China*

*^2^Xinhua Hospital affiliated to Shanghai JiaoTong University School of Medicine, Shanghai, China*

*^3^AstraZeneca Global R&D (China) Co. Ltd., Shanghai, China*

**Full Addresses**

Zhichao Wang and Qingfeng Li: 639 Zhizaoju Road, Shanghai, China, 200023 Shanghai, China

Xin Zhang and Xiaojun Yuan: 1665 Kongjiang Rd, 200092 Shanghai, Shanghai, China

Chunyan Li, Yangbo Liu, Xiaoyun Ge, and Jiajia Zhao: 88 Xizang North Road, JingAn District, 201203 Shanghai, China

**^#^Co-first authors; Zhichao Wang and Xin Zhang contributed equally and share first authorship**

***Ex-employee of AstraZeneca**

**Corresponding authors:** Qingfeng Li and Xiaojun Yuan

Qingfeng Li

639 Zhizaoju Road, Shanghai, China, 200023 Shanghai, China

Email: [dr.liqingfeng@shsmu.edu.cn](mailto:)

Xiaojun Yuan

1665 Kongjiang Rd, 200092 Shanghai, Shanghai, China

Email: [yuanxiaojun@xinhuamed.com.cn](mailto:)

## **SUPPLEMENTARY MATERIALS**

## **1 METHODS**

### **1.1 Study design and patients**

A measurable PN was defined as a PN of ≥3 cm measured in one dimension, which could be seen on ≥3 imaging slices and had a reasonably well-defined contour. Inoperable PN was defined as PN that could not be completely surgically removed without a risk of substantial morbidity due to encasement of, or close proximity to, vital structures, invasiveness, or high vascularity of the PN.

All patients received oral selumetinib on a continuous schedule, where each cycle was 28 days. Dosing continued until disease progression or unacceptable drug-related toxicity.

Target PN were selected by the investigator and were defined as the most clinically relevant PN that was also measurable and either typical or nodular. Additional clinically relevant PN could be selected as a non-target PN. Only one non-target lesion could be selected.

Secondary endpoints included efficacy, health-related quality of life (HRQoL), pain, and physical functioning. Patient’s Global Impression of Change (PGIC) and Patient’s Global Impression of Severity (PGIS) were exploratory endpoints (**Figure 1**).

AEs were coded using the Medical Dictionary for Regulatory Activities (MedDRA) version 25.0 and were graded according to Common Terminology Criteria for Adverse Events (CTCAE) version 5.0. AEs were considered treatment-emergent if they had an onset date on or after the first dose and within 30 days of the last dose, or had worsening of pre-existing events on or after the first dose and within 30 days after the last dose. Treatment-related AEs were assessed by the investigator.

Laboratory data, vital signs, as well as height, weight, and body surface area, electrocardiogram (ECG), echocardiogram (ECHO), ophthalmologic examinations, performance status, bone growth monitoring (pediatric cohort only), and Tanner staging (pediatric cohort only) assessments were performed at screening and at the end of each cycle up to Cycle 4, then every two cycles from Cycle 4 onwards.

Intensive PK samples for single dose measurements were collected from patients before and after first dose of selumetinib 25 mg/m^2^ at Cycle 0 Day 1 until pre-dose at Cycle 1 Day 1. Blood PK samples were taken from patients on Cycle 0 Day 1 within 10 minutes of pre-dose, and then 0.5, 1, 1.5, 3, 6, 8, 12, 24, and 30 hours post dose. On Cycle 1 Day 8, blood PK samples were collected. Intensive PK samples were collected on Cycle 1 Day 8 in dose interval for assessment of PK profile at a steady state. Key PK parameters included time to reach maximum plasma concentration (T_max_); maximum plasma concentration (C_max_); area under the concentration–time curve (AUC) from time 0–12 hours (AUC_0–12h_), from zero to the last measurable concentration (AUC_last_), and from zero to infinity (AUC_inf_); terminal half-life (t_1/2_λ_z_); accumulation ratio based on AUC_(0–12)_ (R_ac_AUC); accumulation ratio based on C_max_ (R_ac_C_max_); and temporal change parameter (TCP) in systemic exposure.

#### **1.2 Inclusion and exclusion criteria**

#### **1.2.1 Inclusion criteria**

Patients were eligible to be included in the study only if all of the following inclusion criteria applied:

- Informed consent
- Capable of giving signed informed consent which included compliance with the requirements and restrictions listed in the Informed Consent Form (ICF) and in this Clinical Study Protocol. For the pediatric cohort, parent/legal guardian consent was required
- Provision of signed and dated written ICF prior to any mandatory study-specific procedures, sampling, and analyses
- Pediatric cohort: mandatory provision of signed and dated parent/legal guardian consent for the study along with the pediatric assent form, when applicable. For patients who reached the age of legal consent during the clinical study, notification was to be required and a new consent form needed to be signed by the patient
- Pediatric cohort: Chinese patients ≥3 years and <18 years of age with a body surface area ≥0.55 m^2^ at the time of study enrollment
- Able to swallow whole capsules. There must be a minimum of six patients each in the 3–11 and 12–17 years age groups at the time of enrollment
- Adult cohort: Chinese patients ≥18 years of age at the time of study enrollment who were able to swallow whole capsules
- Diagnosed with (i) neurofibromatosis type 1 (NF1) per National Institutes of Health (NIH) Consensus Development Conference Statement 1988^17^ and (ii) inoperable plexiform neurofibroma (PN). In addition to PN, patients must have ≥1 other diagnostic criterion for NF1 (NIH Consensus Development Conference Statement 1988):
- Six or more café-au-lait macules >5 mm in greatest diameter in pre-pubertal individuals and >15 mm in greatest diameter in post-pubertal individuals
- Freckling in the axillary or inguinal regions
- Optic glioma
- Two or more Lisch nodules (iris hamartomas)
- A distinctive osseous lesion such as sphenoid dysplasia or tibial pseudarthrosis
- A first-degree relative with NF1

A PN is defined as a neurofibroma that has grown along the length of a nerve and may involve multiple fascicles and branches. A histologic confirmation of the tumor was not necessary in the presence of consistent clinical and radiographic findings, but was to be considered if malignant degeneration of a PN was clinically suspected.

Inoperable PN is defined as PN that cannot be completely surgically removed without a risk of substantial morbidity due to encasement of, or close proximity to, vital structures, invasiveness, or high vascularity of the PN.

- Required treatment due to actual symptoms or had the potential to develop significant clinical complications, as judged by the investigator
- Patients must have ≥1 measurable typical or nodular PN, defined as a lesion of ≥3 cm measured in one dimension, which could be seen on ≥3 imaging slices and had a reasonably well-defined contour
- Patients who have undergone surgery for resection of a PN were eligible provided the PN was incompletely resected and was measurable. The target PN was defined as the clinically most relevant PN, which had to be amenable to volumetric magnetic resonance imaging (MRI) analysis and classified as either typical or nodular (i.e. must not be solitary nodular)
- Patients >16 years of age: Karnofsky performance level of ≥70
- Children ≤16 years old must have a Lansky performance of ≥70
- Patients who were wheelchair bound because of paralysis secondary to a PN were considered ambulatory when they were in their wheelchair. Similarly, patients with limited mobility secondary to a need for mechanical support (such as an airway PN requiring tracheostomy or continuous positive airway pressure) were also considered ambulatory for the purpose of the study
- Adequate hematologic function defined as absolute neutrophil count ≥1.5×10^9^/L, hemoglobin ≥9 g/dL, and platelet count ≥100×10^9^/L. Patient must be without growth factor support and platelet transfusion support 7 days before the screening assessment
- Adequate organ function defined as follows: aspartate aminotransferase and alanine aminotransferase ≤2× upper limit of normal (ULN), total bilirubin ≤1.5× ULN except in the case of patients with documented Gilbert’s disease (≤2.5× ULN). Estimated creatinine clearance of ≥60 mL/minute, calculated using the formula of Cockcroft and Gault ([140 minus age] • mass [kg]/[72 • creatinine mg/dL] • multiply by 0.85 if female) or normal serum creatinine based on age, as described below:

| Age (years) | Maximum creatinine (mg/dL) | Maximum creatinine (µmol/L) |
| --- | --- | --- |
| ≤5 | 0.8 | 70.7 |
| >5 to ≤10 | 1.0 | 88.4 |
| >10 to ≤15 | 1.2 | 106.1 |
| >15 | 1.5 | 132.6 |

- Negative pregnancy test (urine or serum) for female patients of childbearing potential
- A female of childbearing potential was defined as a patient ≥9 years of age or those showing pubertal development
- Female patients with childbearing potential must be 1 year post-menopausal (amenorrheic for the past 12 months without an alternative medical cause), surgically sterile, or using an acceptable method of contraception for the duration of the study (from the time of signing consent) and for ≥4 weeks after the last dose of selumetinib to prevent pregnancy
- An acceptable method of contraception was a combination of two methods, including but not limited to implants, injectables, combined oral contraceptives (e.g. the contraceptive pill), some intrauterine devices (e.g. placement of an intrauterine device or intrauterine system), and a male partner with sterilization. True sexual abstinence was also an acceptable method of contraception. Reliable methods of contraception/birth control should be used consistently and correctly. The following age-specific requirements must also apply:
  - Women <50 years old were considered post-menopausal if they had been amenorrheic for the past 12 months or more following cessation of exogenous hormonal treatments. The levels of luteinizing hormone and follicle-stimulating hormone must also be in the post-menopausal range
  - Women ≥50 years old were considered post-menopausal if they had been amenorrheic for the past 12 months or more following cessation of all exogenous hormonal treatments, had had radiation-induced oophorectomy with the last menses >1 year ago, had had chemotherapy-induced menopause with >1 year interval since the last menses, or had had surgical sterilization by either bilateral oophorectomy or hysterectomy

#### **1.2.2 Exclusion criteria**

- Evidence of malignant peripheral nerve sheath tumor
- Prior malignancy (except for adequately treated basal cell or squamous cell skin cancer, in situ cervical cancer, or other cancer from which the patient had been disease-free for ≥2 years or which would not have limited survival to <2 years) or other cancer requiring treatment with chemotherapy or radiation therapy. Note: these cases must have been discussed with the study physician
- A life-threatening illness, medical condition, or organ system dysfunction, which in the investigator's opinion, could compromise the patient’s safety, interfere with the absorption or metabolism of selumetinib, or put the study outcomes at undue risk
- Patients with clinically significant cardiovascular disease as defined by the following:
  - Known inherited coronary disease
  - Blood pressure (BP):
    - For pediatric patients, BP > the 95th percentile for age, height, and gender measured
    - Uncontrolled hypertension (at screening: BP ≥150/95 despite optimal therapy)
  - Acute coronary syndrome within 6 months before starting treatment
  - Uncontrolled angina – Canadian Cardiovascular Society Grade II–IV despite medical therapy
  - Symptomatic heart failure New York Heart Association Class II–IV, prior or current cardiomyopathy, or severe valvular heart disease
  - Prior or current cardiomyopathy including but not limited to the following:
    - Known hypertrophic cardiomyopathy
    - Known arrhythmogenic right ventricular cardiomyopathy
    - Previous moderate or severe impairment of left ventricular ejection fraction (LVEF) <45% on echocardiogram or equivalent on multigated acquisition (MUGA) even if full recovery has occurred
  - Baseline LVEF below the lower limit of normal (LLN) or <55% measured by echocardiogram or institution’s LLN for MUGA
  - Severe valvular heart disease
  - Current or history of atrial fibrillation
  - QT interval corrected by Fridericia’s method (QTcF) >450 ms or other factors that increased the risk of QT prolongation
- Known history of human immunodeficiency virus, serologic status reflecting active hepatitis B virus (HBV) or hepatitis C virus (HCV) infection, or any uncontrolled active systemic infection:
  - Patients who were hepatitis B core antibody positive and who were hepatitis B surface antigen (HBsAg) negative would need to have a negative HBV deoxyribonucleic acid polymerase chain reaction (PCR) result before enrollment
  - Patients who were HBsAg positive or hepatitis B PCR positive were to be excluded
  - Patients who were hepatitis C antibody positive would need to have a negative HCV ribonucleic acid PCR result before enrollment. Those who were hepatitis C PCR positive were to be excluded
- Patients with the following ophthalmological findings/conditions:
  - Current or past history of retinal pigment epithelial detachment/central serous retinopathy or retinal vein occlusion
  - Intraocular pressure (IOP) >21 mmHg (or ULN adjusted by age) or uncontrolled glaucoma (irrespective of IOP). Patients with known glaucoma and increased IOP who did not have meaningful vision (light perception only or no light perception) and were not experiencing pain related to the glaucoma, could be eligible after discussion with the study physician
  - Any other significant abnormality on ophthalmic examination that would make the patient unsuitable for enrollment into the study, as assessed by the investigator
  - Ophthalmologic findings secondary to long-standing optic pathway glioma (such as visual loss, optic nerve pallor or strabismus) or longstanding orbito-temporal PN (such as visual loss, strabismus) would NOT be considered a significant abnormality for the purposes of the study
- Had received or were receiving an investigational product (IP) or other systemic PN target treatment (including chemotherapy, hormonal therapy, radiation therapy, immunotherapy, or biologic therapy) within 4 weeks before the first dose of study treatment, or within a period during which the IP or systemic PN target treatment has not been cleared from the body (e.g. a period of 5 ‘half-lives’), whichever was the most appropriate as judged by the investigator
- Inability to undergo MRI and/or contraindication for MRI (i.e. prosthesis or orthopedic or dental braces that would interfere with volumetric analysis of target PN on MRI)
- Had refractory nausea and vomiting, chronic gastrointestinal diseases (e.g. inflammatory bowel disease), or significant bowel resection that would adversely affect the absorption/bioavailability of the orally administered study medication
- Have had prior treatment with a MEK, RAS, or RAF inhibitor (including, but not limited to, vemurafenib)
- Supplementation with vitamin E greater than 100% of the daily recommended dose. Any multivitamin containing vitamin E must have been stopped before initiation of selumetinib
- Receiving herbal supplements or medications known to be strong inhibitors or inducers of the cytochrome P450 (CYP)2C19 and CYP3A4 enzymes unless such products can be safely discontinued ≥14 days before the first dose of study medication
- Had any unresolved chronic toxicity except alopecia with Common Terminology Criteria for Adverse Events (CTCAE) Grade ≥2, from previous anticancer therapy including radiation
- Had recent major surgery within a minimum of 4 weeks before starting study treatment, with the exception of surgical placement for vascular access. Had planned major surgery during the treatment period
- Known severe hypersensitivity to selumetinib or any excipient in the selumetinib formulation, or history of allergic reactions attributed to compounds of similar chemical or biologic composition to selumetinib
- Breastfeeding or pregnant (it was allowed to discontinue lactation and participate in the study; however, resumption of lactation after study completion was not allowed)
- Had evidence of any other significant clinical disorder or laboratory finding that, as judged by the investigator, made it undesirable for the patient to participate in the study

Judgment by the investigator that the patient was not to participate in the study if the patient was unlikely to comply with study procedures, restrictions, and requirements

### **1.3 Objectives and Assessments**

#### 1.3.1 Plexiform neurofibroma response

PN were assessed at screening, Cycle 4, then every four cycles up to the end of Cycle 24, and every six cycles thereafter, using magnetic resonance imaging (MRI). PN response was assessed by the investigator and independent central review (ICR) according to the Response Evaluation in Neurofibromatosis and Schwannomatosis (REiNS) criteria.^1^ Objective response rate (ORR) was defined as the proportion of patients who had a complete response (CR) or confirmed partial response (cPR; defined as a target PN volume decrease ≥20% compared with baseline, confirmed by a consecutive scan within 3–6 months after first response). Duration of response (DoR) was defined as the time from the date of first documented response, which is subsequently confirmed until the date of documented progression or death in the absence of progression. Progressive disease (PD) was defined as a ≥20% increase in target PN volume relative to baseline or the best overall response (maximum tumor reduction) recorded after partial response. Progression-free survival (PFS) was defined as the time from the date of the first dose until progression, or death due to any cause. Time to progression (TTP) was defined as the time from the date of the first dose until progression. Time to response (TTR) was defined as the time from the date of the first dose until the date of the first documented response, which is subsequently confirmed. DoR and TTR analyses included patients who had reached CR or cPR by primary data cut-off.

#### 1.3.2 Clinical outcome and health-related quality of life

Pain intensity in adult patients was measured by Numeric Rating Scale (NRS)-11.^2^ Physician-selected tumor pain, overall tumor pain, and overall pain were measured on a scale from 0 to 10, where 10 was the worst pain. Pain intensity was measured in pediatric patients aged 4–17 years old using the Faces pain scale.^3^ Patients self-reported the worst pain experienced in the last 2 weeks at each cycle on a scale of 0–10.

Pain interference was measured in both adult and pediatric patients using Pain Interference Index (PII), which is based on a 7-point Likert scale, where 0 is no interference, and 6 is complete interference with daily activities.^4,5^ PII scores were self-reported by adult patients and pediatric patients aged 8–17 years, as well as caregiver-reported in the pediatric patients aged 5–17 years.

Patient-Reported Outcomes Measurement Information System (PROMIS) Physical Functioning Scales were used to evaluate physical function, by evaluating mobility and upper extremity function in the pediatric cohorts.^6^ In the pediatric cohort, self-reported results were evaluated for patients aged 8–17 years, and caregiver-reported results were evaluated for patients aged 5–17 years. Adult physical functioning was assessed by PROMIS short form.

In the adult cohort, HRQoL was evaluated using European Organisation for Research and Treatment of Cancer Quality of Life Questionnaire Core 30 (EORTC QLQ-C30) and Plexiform Neurofibromas Quality of Life scale (PlexiQoL).^7,8^ EORTC QLQ-C30 comprised 30 questions covering functional and symptom scales, as well as global measure of health status and additional common symptoms; a score between 0 and 100 was derived from each scale, where higher scores on global health status and QoL indicated better health status/function, but higher scores on the symptom scales represented greater symptom severity. The PlexiQoL consisted of 18 items with a total score from 0 to 18, where higher scores represented worse QoL. In the pediatric cohort, HRQoL was evaluated using Pediatric Quality of Life Inventory (PedsQL), using both self-reported (5–17 years old) and caregiver-reported results (3–17 years old). PedsQL consists of four subscales (physical, emotional, social, and school functioning).^9^ For self-reported and caregiver-reported PedsQL, items were reverse-scored and linearly transformed to a 0−100 scale, where 100 is never a problem and 0 is almost always a problem; higher scores represented better QoL. The Total Scale Score was the average across all items answered across all four scales. Patients aged 5–17 years completed PedsQL questionnaires, as well as caregivers of all pediatric patients.

PGIC and PGIS were measured by self-report in the adult cohort, and by self- (8–17 years old) and caregiver-report (≤17 years old) in the pediatric cohort. PGIC was used to evaluate the patients’ global impression of change on a 7-point scale, where 1 was much improved and 7 was much worse compared with how they felt before selumetinib initiation. PGIS was used to evaluate the severity of symptoms experienced by a patient on a 6-point scale, where 0 is no symptoms and 5 is very severe symptoms. For both measures, tumor pain, overall pain, and tumor-related problems were scored.

Clinical outcome assessments were performed at screening and at the end of each cycle until the end of Cycle 4, every two cycles up to the end of Cycle 12, every four cycles up to the end of Cycle 24, and every six cycles thereafter, except for PGIC, which was first measured at Cycle 1.

### **1.4 Data and statistical analysis**

Descriptive statistics were used for all outcomes, and no formal hypothesis testing was used in this study. SAS^®^ version 9.4 or above was used for all analyses. Demographic data, baseline patient characteristics, efficacy, clinical outcome assessment, exposure, and safety data were analyzed for the safety analysis set. The PK data were summarized based on the PK analysis set, and PK parameters were computed using non-compartmental methods with Phoenix^®^ WinNonlin^®^ version 8.1.1.Median DoR, TTR, PFS, and TTP were calculated using the Kaplan–Meier method. Primary analysis of clinical outcome assessments was based on descriptive statistics, including change from baseline. Change from baseline for pain, physical functioning, and HRQoL measures were analyzed using a mixed model repeated measures (MMRM) approach, where there were ≥10 patients per visit.

**2 RESULTS**

**2.1 Baseline characteristics and demographics**

Key baseline demographics and clinical characteristics are shown in **Table 1**.

*2.1.1 Adult*

Within the adult cohort, the most common target PN location was at extremity (n=6; 38%). The median target PN volume was 691.7 mL (range 46.2–7746.3 mL), and six patients had non-target PN. Overall, 81% of adult patients had previously received ≥1 prior NF1 or PN treatment. The median (range) actual and total treatment durations were 13.1 (7.5−19.4) months and 13.2 (8.6−19.4) months, respectively.

*2.1.2 Pediatric*

Within the pediatric cohort, the most common target PN location was at extremity (n=5; 31%). The median target PN volume was 517.4 mL (range 47.6–2664.4 mL), and one patient had non-target PN. Most patients (75%) had previously received ≥1 prior NF1 or PN treatment. In the pediatric cohort, the median (range) actual and total treatment durations were 11.3 (10.1−18.9) months.

**2.2 Safety**

*2.2.1 Adult*

The most common AE in the adult cohort was dermatitis acneiform (n=13; 81%); all occurrences of this AE were considered treatment-related. Three (19%) patients experienced AEs that led to dose modification: one (6%) experienced an AE that resulted in dose reduction and two (13%) had dose interruption because of AEs.

*2.2.2 Pediatric*

The most common AE was pyrexia, which was reported in six (38%) patients; in one (6%) patient, this was considered treatment-related. One patient experienced ≥1 Grade 3 AE: this patient reported two Grade 3 SAEs of sepsis and one Grade 3 SAE of urinary tract infection, which led to selumetinib interruption; however, these were not considered to be related to selumetinib treatment. These were the only SAEs reported in the pediatric cohort.

**2.3 Pharmacokinetics**

PK parameters of selumetinib are provided in **Table 3**. PK parameters of N-desmethyl selumetinib are provided in **Table S1**. The geometric mean plasma concentration–time profiles are shown in **Figure S1**.

*2.3.1 Adult*

Selumetinib showed a time-independent PK characteristic with the systemic exposure TCP of AUC ≤1.13 in the adult cohort. The geometric means of C_max,ss_ (ng/mL) and AUC_0-12,ss_ (hours*ng/mL) were 1168 and 3932, respectively.

*2.3.2 Pediatric*

Selumetinib demonstrated a time-independent PK characteristic with the systemic exposure TCP of AUC ≤1.26 in the pediatric cohort. The geometric means of C_max,ss_ (ng/mL) and AUC_0-12,ss_ (hours*ng/mL) were 1032 and 2961, respectively.

**2.4 Efficacy**

Response and PN volume change were assessed by ICR and investigator as per REiNS criteria and are shown in **Table S2**. Best percentage changes from baseline in the target PN volume are shown in **Figure S2**.

*2.4.1 Adult*

In the adult cohort, all 16 patients were evaluable for response. An ORR of 19% and 25% was achieved per ICR and investigator assessment, respectively. Based on ICR and investigator assessment, three (19%) and four (25%) patients, respectively, achieved cPR, although no patient achieved CR by either assessment. A best reduction of ≥20% in target PN volume from baseline was reported in eight (50%) patients per ICR and five (31%) patients per investigator (**Figure 1A and B; Figure S3A and B**). Of the six adult patients with non-target PN, all were non-progressive by the primary data cut-off per both ICR and investigator assessment, with a mean best percentage change (standard deviation [SD]) of −33% (±24.2%) by ICR and −34.8% (±40.4%) by investigator assessment (**Figure S4A and B**). The median DoR was not reached at this cut-off per ICR and investigator assessment. The TTR was 7.7 months and 3.8 months based on ICR and investigator assessment, respectively. The concordance between ICR- and investigator-assessed target PN response was 63% for adult patients. The median PFS, and TTP were not reached at primary data cut-off per both ICR and investigator assessment.

*2.4.2 Pediatric*

All 16 patients in the pediatric cohort were evaluable for response. An ORR of 31% and 63% was achieved per ICR and investigator assessment, respectively. Based on ICR and investigator assessments, five (31%) and 10 (63%) patients, respectively, achieved cPR, although no pediatric patient achieved CR by either assessment. A best reduction of ≥20% from baseline was observed in 12 patients (75%) per ICR and 14 patients (88%) per investigator assessment (**Figure 1C and D; Figure S3C and D**). The non-target PN reported in one pediatric patient was non-progressive by data cut-off per ICR and investigator assessment with a best percentage change of −23% by ICR and −36.9% by investigator (**Figure S4C and D**). The median DoR was not reached for the pediatric cohort at primary data cut-off per ICR and investigator assessment. Based on ICR and investigator assessments, the median TTR was 3.8 and 3.7 months, respectively. The concordance between ICR- and investigator-assessed target PN response was 50% for pediatric patients. Median PFS was 15.9 months per ICR assessment but was not reached based on investigator assessment after a median follow-up time of 11.0 months. Based on ICR assessment, the median TTP was 15.9 months; however, based on investigator assessment, median TTP was not reached.

**2.5 Pain**

*2.5.1 Adult*

Self-reported NRS-11 (physician-selected tumor pain) scores in the adult cohort did not indicate any meaningful differences in pain intensity over time, and baseline pain generally remained consistent until Cycle 10, which was supported by the MMRM analysis (**Figure S5A**). All four patients who had an NRS-11 score ≥2 at baseline experienced a decrease in pain intensity as early as Cycle 1. No patient had received any pain medication one week before the first dose of selumetinib; two patients received pain medication for a short time due to pain caused by AEs during study treatment.

PII scores indicated that there were no meaningful differences in the extent to which pain interfered with daily activities. Generally, the baseline scores that patients reported were maintained throughout. A slight improvement/general maintenance was observed in pain interference with daily activities post selumetinib. MMRM analysis supported these results (**Figure S6A**).

*2.5.2 Pediatric*

Pain intensity was measured in pediatric patients using the Faces pain scale. At baseline, 56% of patients had a pain intensity score of 0, with the remaining patients reporting a score of 2. By primary data cut-off, there was a trend of decreasing pain intensity by this assessment. MMRM analysis also indicated an overall decrease between baseline and Cycle 12 (**Figure S5B**).

Pain interference was measured using PII by self- and caregiver-report in all but one patient in the pediatric cohort. The interference of pain on daily function showed a trend of decreasing over time, with some fluctuation, based on an adjusted mean negative change compared with baseline throughout Cycle 12 for both self-reported and caregiver-reported outcomes. This trend was supported by MMRM analysis (**Figure S6B and C**).

**2.6 Physical functioning**

*2.6.1 Adult*

Physical functioning of adult patients was measured using PROMIS short form with a total score of 40. The mean score at baseline for PROMIS short form was 38.2. There was a slight decrease in PROMIS physical function scores based on adjusted mean change from baseline by MMRM analysis (**Figure S7A**). Two patients who had reported baseline scores below 40, reported improved scores in physical function at data cut-off.

*2.6.2 Pediatric*

Physical functioning (mobility and upper extremity) in pediatric patients was measured using both self and caregiver reports from PROMIS. There was a trend of improvement in both self- and caregiver-reported physical functioning from baseline. The MMRM analysis indicated a slight improvement in both self- and caregiver-reported scores for mobility and upper extremity (**Figure S7B−E**).

**2.7 Health-related quality of life**

*2.7.1 Adult*

For adult patients, HRQoL was measured by PlexiQL and EORTC QLQ-C30, which were both self-reported. The mean change in PlexiQL total score showed a trend of improvement in HRQoL compared with baseline. The adjusted mean change of PlexiQL total score by MMRM analysis also indicated a small improvement at all post-baseline time points (**Figure S8A**). EORTC QLQ-C30 scores demonstrated a generally stable trend throughout the study period.

*2.7.2 Pediatric*

Both self- and caregiver-reported PedsQL total and subscale scores showed a trend of continuous improvement over the first four cycles, and the improvement in total score and subscale score at Cycle 4 was generally maintained until data cut-off. Improvements in QoL were reflected in the MMRM analysis (**Figure S8B and C**).

**2.8 Patient’s global impression of change and patient’s global impression of severity**

*2.8.1 Adult*

Most adult patients reported an improvement in overall pain, tumor pain, and tumor-related problems throughout the study post-selumetinib treatment by the primary data cut-off. No patients reported worsening of PGIC post treatment (**Figure S9A**).

The percentage of patients that reported very mild to no symptoms in tumor pain was relatively maintained at both Cycle 4 (13/15; 87%) and 8 (12/15; 80%), compared with baseline assessments (13/16; 81%) (**Figure S10A**). The percentage of patients that reported very mild to no symptoms in tumor-related problems was also generally maintained at both Cycle 4 (13/15; 87%) and 8 (12/15; 80%), compared with baseline assessments (13/16; 81%) (**Figure S10A**).

*2.8.2 Pediatric*

Most pediatric patients reported improvements in overall pain, tumor-related pain, and tumor-related problems throughout treatment, compared with baseline, and no patients reported worsening of PGIC post treatment. These trends were consistent among both self- and caregiver-reported results for PGIC (**Figure S9B and C**).

For both self- and caregiver-reported PGIS measures, the percentage of patients reporting no symptoms was higher than baseline at almost every time point after receiving selumetinib (**Figure S10B and C**).

### **3 DISCUSSION**

Selumetinib is a selective MEK1/2 inhibitor approved for the treatment of pediatric patients with NF1 and symptomatic, inoperable PN by the FDA (≥2 years), the EMA (≥3 years),^18,20^ and regulatory bodies in Japan and China (≥3 years).^21,22^ Based on data from this Phase 1 study, selumetinib (25 mg/m^2^ twice daily) was generally well tolerated with an acceptable benefit–risk profile in adult and pediatric patients with NF1-PN in China. Specifically, this was the first study in China to present data on the safety of adult patients with NF1-PN. The safety profile reported here was in line with the known safety profile of selumetinib; no new safety concerns were identified. Most AEs in this study were mild in severity; the majority were Grade 1/2 in both pediatric and adult cohorts. Only two adult patients and one pediatric patient reported Grade ≥3 AEs; this is similar to that reported in both the Phase 1 and 2 parts of SPRINT, with most AEs Grade 1 or 2.^16,19^ Dermatitis acneiform was the most commonly reported AE in the adult cohort (81%), and pyrexia was the most commonly reported AE in the pediatric cohort (38%), all of which were Grade 1/2 events. Dermatitis acneiform and pyrexia have been reported as commonly occurring AEs in previous selumetinib studies.^18,19,33,34^

The PK of selumetinib was generally consistent between the two cohorts, with rapid absorption and no significant accumulation after multiple doses. The PK profile of selumetinib observed here in Chinese pediatric patients was similar to that seen in SPRINT^16^ and in a Phase 1 Japanese trial.^35^ The C_max_ (870.9 ng/mL) reported after a single dose was comparable to the C_max_ reported in Phase 2 SPRINT (825 ng/mL).^19^ The median (range) T_max_ for single dose (1.5 [1.0–3.0]) and steady state (1.5 [0.5–3.0]) observed in this study showed similarity with the median (range) T_max_ published for White (1.0 (1.0–4.0)] and Asian [1.0 (1.0–4.0)] patients in a pooled analysis of healthy individuals.^36^

Selumetinib demonstrated promising efficacy in both the adult and the pediatric cohorts, with almost all patients experiencing a reduction in target PN volume as best response. In this study, the efficacy of selumetinib in the adult cohort was reported for the first time in Chinese patients; a best reduction of ≥20% from baseline was observed in eight (50%) patients per ICR and five (31%) patients per investigator assessment. In the pediatric cohort, this reduction was observed in 12 (75%) patients per ICR and 14 (88%) patients per investigator assessment. The concordances between ICR- and investigator-assessed target PN responses observed in the adult and pediatric cohorts were 63% and 50%, respectively, indicating a discrepancy between efficacy results based on the two parties’ assessments. A similar trend was seen in SPRINT between the ORR reported by the investigator and ICR (66% vs 44%, respectively).^18^ Differences in categorizations of best objective response between the investigator and ICR analyses were primarily in the assignment of unconfirmed PR (uPR) versus cPR and stable disease in the adult cohort, and uPR versus cPR in the pediatric cohort. There are several reasons why this may have happened. Investigators are likely to have had access to the actual status of each patient, therefore it may be expected that these results more accurately reflect the real-life situation. Additionally, as REiNS is a relatively new criterion, no consensus on range of discrepancy has been established. Regardless, in both cohorts, a trend of tumor volume reduction was consistently observed from both investigator and ICR assessments.

Patients with NF1-PN often experience a range of associated morbidities, including pain, disfigurement, and internal organ compression, which can negatively impact overall physical functioning and QoL.^9-11^ Here, improvements from baseline in both pain intensity and interference, and physical functioning were reported in the pediatric cohort. Notably, no patients reported worsening of symptoms post treatment. Results in the adult cohort varied from the pediatric cohort, as no meaningful differences in pain intensity were observed over time and a slight worsening in physical functioning from baseline was reported, although an improvement in pain interference over time was observed. Overall improvements in HRQoL were observed in both cohorts. In previous studies, a minimal clinically important difference (MCID) had been defined for QoL measures. For example, in SPRINT, changes in PedsQL greater than half SD (>8.7 and >8.1 for self- and caregiver-reported results, respectively) were defined as the MCID.^19^ By applying these thresholds, it was determined that approximately half the patients in SPRINT had clinically meaningful increases in PedsQL after a year of selumetinib treatment.^19^ Although no MCIDs were established within the current study, continuous improvement of QoL in pediatric patients was observed during selumetinib treatment. Two measures were used to assess HRQoL in the adult cohort; the PlexiQoL measure indicated an improvement in HRQoL, whereas the EORTC QLQ-C30 measure indicated stability in HRQoL throughout the study.

Improvements in PGIC measures were observed in the majority of adult and pediatric patients in this study; improvements in PGIC were also reported in pediatric patients who had been receiving selumetinib for a year in SPRINT.^19^ In regards to PGIS measures, while the percentage of adult patients reporting no/very mild symptoms in each category relatively remained unchanged at Cycles 4 and 8, a greater number of pediatric patients reported no symptoms at most time points after they started selumetinib treatment compared with baseline.

Several limitations have been noted for this study. First, this was a single-arm trial, in which a small number of patients were enrolled. Although there are no natural history studies in China that can be used for comparison with these data, the results presented here are consistent with those reported previously for pediatric patients in SPRINT, for which a natural history comparison was published.^19^ Second, this study had a relatively short duration of follow-up in comparison to SPRINT.^19^ The impact of the COVID-19 pandemic on the study must also be noted; some adult and pediatric patients failed to complete scheduled tumor assessment visits according to the study plan, resulting in missed or delayed MRI scans potentially impacting efficacy results. However, it is essential to emphasize that despite these delays, subsequent follow-up scans did not reveal any significant impact on the overall conclusion of the study.

Overall, this study confirmed that selumetinib demonstrates promising efficacy in both adult and pediatric patients with NF1-PN at the primary data cut-off, with most patients experiencing a reduction in target PN volume and an acceptable benefit–risk profile, with improvements in HRQoL. For these reasons, selumetinib may address the unmet medical need for patients with NF1 and inoperable PN in China.

## **4 SUPPLEMENTARY FIGURES AND TABLES**

TABLE S1 PK parameters of N-desmethyl selumetinib following single and multiple doses.

| **N-desmethyl selumetinib** | **Single dose** | | **Steady state** | |
| --- | --- | --- | --- | --- |
|  | **Adult (n=16)** | **Pediatric (n=16)** | **Adult (n=15)** | **Pediatric (n=16)** |
| T_max_, h, median (range) | 1.0  （0.9–1.6） | 1.5  （1.0–3.0） | 1.5  （1.5–1.6） | 1.5  （0.5–3.0） |
| C_max_, ng/mL, GeoMean (GCV%) | 84  （32.29%） | 64  （72.72%） | 75  （29.21%） | 77  （32.46%） |
| AUC_0-12h_, ng*h/mL, GeoMean (GCV%) | 237  （26.95%） | 175  （54.72%） | 289  （27.48%） | 252  （36.45%） |
| AUC_last_, ng*h, GeoMean (GCV%) | 267  （31.92%） | 189  （61.72%） | – | – |
| AUC_inf_, ng*h/mL, GeoMean (GCV%) | 310  （29.21%） （n=13） | 252  （48.12%）  （n=13） | – | – |
| t_1/2_λ_z_, h, AriMean ± SD | 7.754±3.387 (n=14) | 7.852±3.378 （n=13） | – | – |
| R_ac_ AUC, AriMean ± SD | – | – | 1.253±0.3049 | 1.515±0.5015 |
| R_ac_ C_max_, AriMean ± SD | – | – | 0.9662±0.3735 | 1.388±0.8317 |
| TCP, AriMean ± SD | – | – | 0.9522±0.2135 （n=12） | 1.107±0.2960 （n=13） |

AriMean, arithmetic mean; AUC_0-12h_, area under the concentration–time curve from time 0 to 12 hours; AUC_inf_, area under the concentration–time curve from zero to infinity; AUC_last_, area under the concentration–time curve from zero to the last measurable concentration; C_max_, maximum plasma concentration; GeoMean, geometric mean; PK, pharmacokinetics; R_ac_ AUC, accumulation ratio based on AUC_(0-12)_, ratio of the AUC_(0-12),ss_ following multiple dosing by the AUC_(0-12)_ following first dose; R_ac_ C_max_, accumulation ratio based on C_max_, ratio of the C_max,ss_ following multiple dosing by the AUC_(0-12)_ following first dose; SD, standard deviation; t_1/2_λz, terminal half-life; TCP, temporal change parameter in systemic exposure, ratio of the AUC_(0-12),ss_ following multiple dosing by the first dose AUC_inf_; T_max_, time to reach maximum plasma concentration.

TABLE S2 Response assessed by ICR and investigator as per REiNS criteria at primary data cut-off (after the last dosed patient completed Cycle 10 Day 28).

| **Response assessment** | **Adult patients  (n=16)** | | **Pediatric patients  (n=16)** | |
| --- | --- | --- | --- | --- |
|  | **ICR** | **Investigator** | **ICR** | **Investigator** |
| **ORR, n (%)** | 3 (19) | 4 (25) | 5 (31) | 10 (63) |
| **Best response, n (%)**  CR  cPR^†^  Unconfirmed PR^‡^  SD  PD  Not evaluable | 0  3 (19)  5 (31)  7 (44)  1 (6)  0 | 0  4 (25)  1 (6)  11 (69)  0  0 | 0  5 (31)  7 (44)  3 (19)  1 (6)  0 | 0  10 (63)  4 (25)  2 (13)  0  0 |
| **Patients with target PN volume reduction,^§^ n (%)** | n=16  14 (88) | n=16 16 (100) | n=16  14 (88) | n=16 16 (100) |
| **Best percentage change from baseline in target PN volume, mean (± standard deviation)** | n=16 −20 (±18) | n=16  −17 (±12) | n=16 −33 (±22) | n=16 −34 (±13) |
| **Median duration of response, months (range)** | Not reached  (8.4–11.0) | Not reached  (7.6–15.4) | Not reached (3.4–12.2) | Not reached (4.7–11.2) |

^†^PR was defined as a decrease in volume of the target PN by ≥20% compared with baseline, a response of non-PD in the non-target PN, and no new lesions.
^‡^PR was considered unconfirmed at the first detection until observed again within 3–6 months, PR achieved but either no confirmation assessment performed or a confirmation assessment performed but response not confirmed.
^§^Best percentage change was derived as the maximum reduction from baseline or the minimum increase from baseline to data cut-off in the absence of reduction.
cPR, confirmed partial response; CR, complete response; ICR, independent central review; ORR, objective response rate; PD, progressive disease; PN, plexiform neurofibroma; PR, partial response; REiNS, Response Evaluation in Neurofibromatosis and Schwannomatosis; SD, stable disease.

FIGURE S1 Geometric mean plasma concentration–time profiles of selumetinib and N-desmethyl selumetinib for the adult (A single dose, B multiple dose) and pediatric (C single dose and D multiple dose) cohorts on a semi-logarithmic scale (PK Analysis Set).

**A**


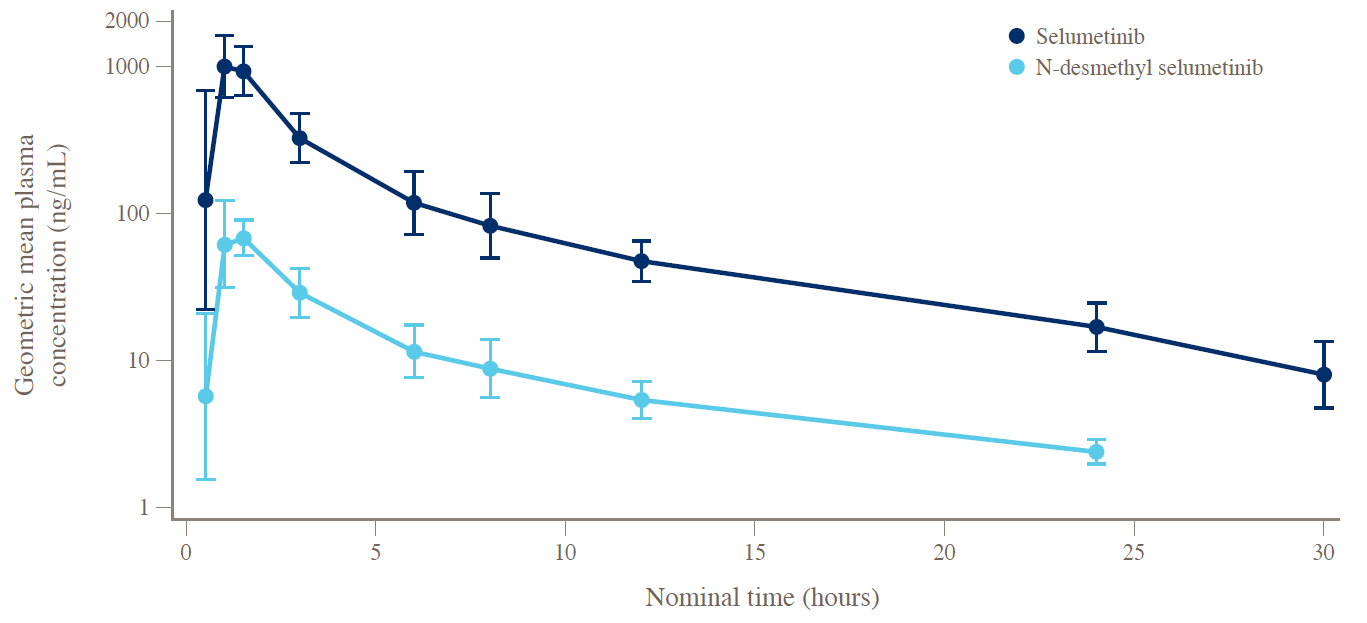


**B**


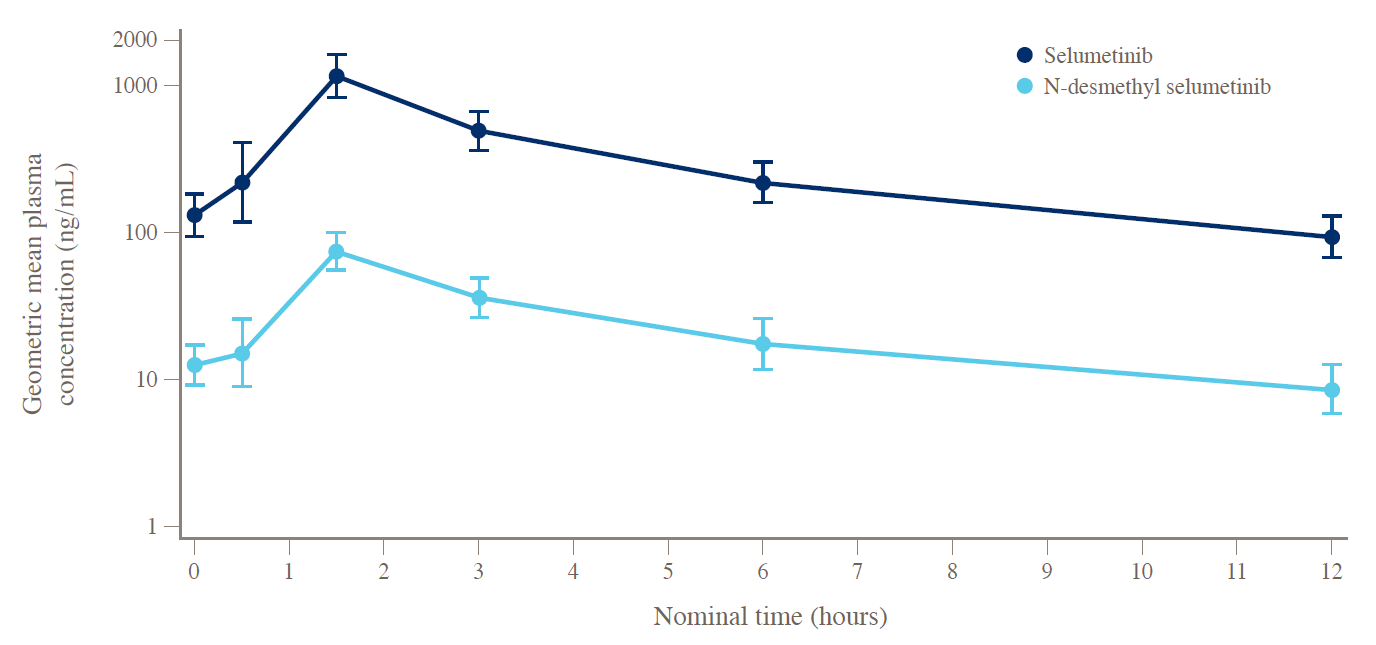


**C**


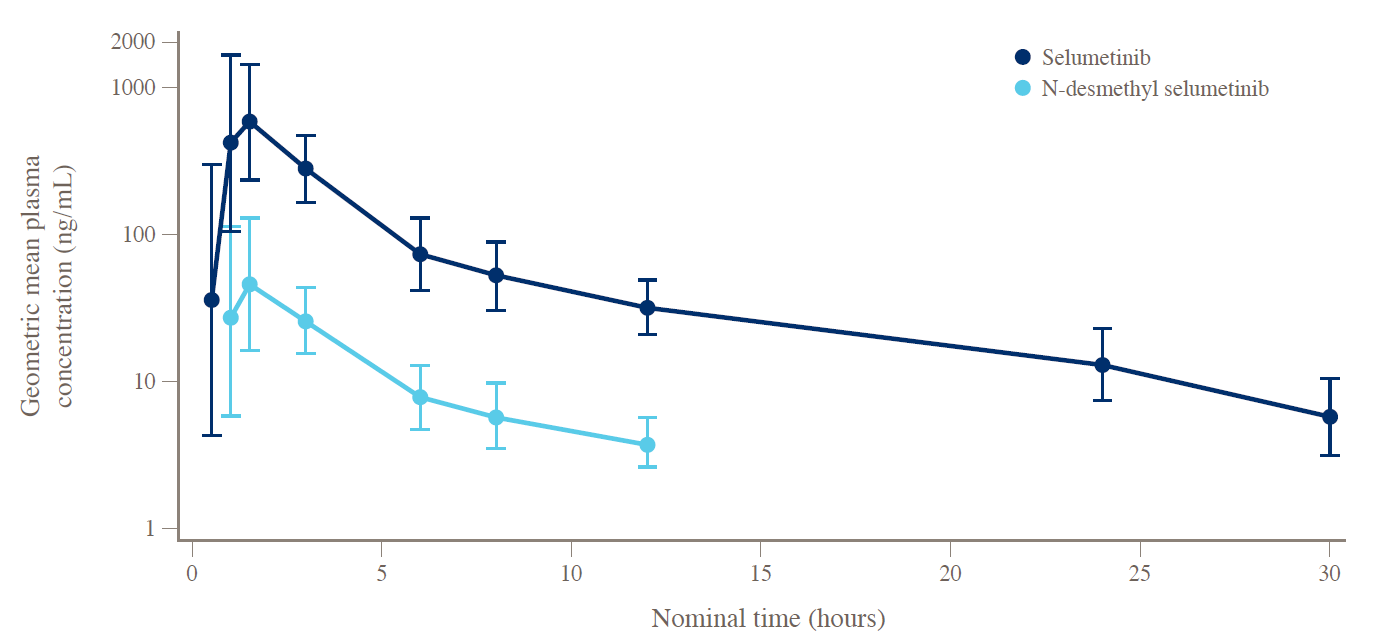


**D**


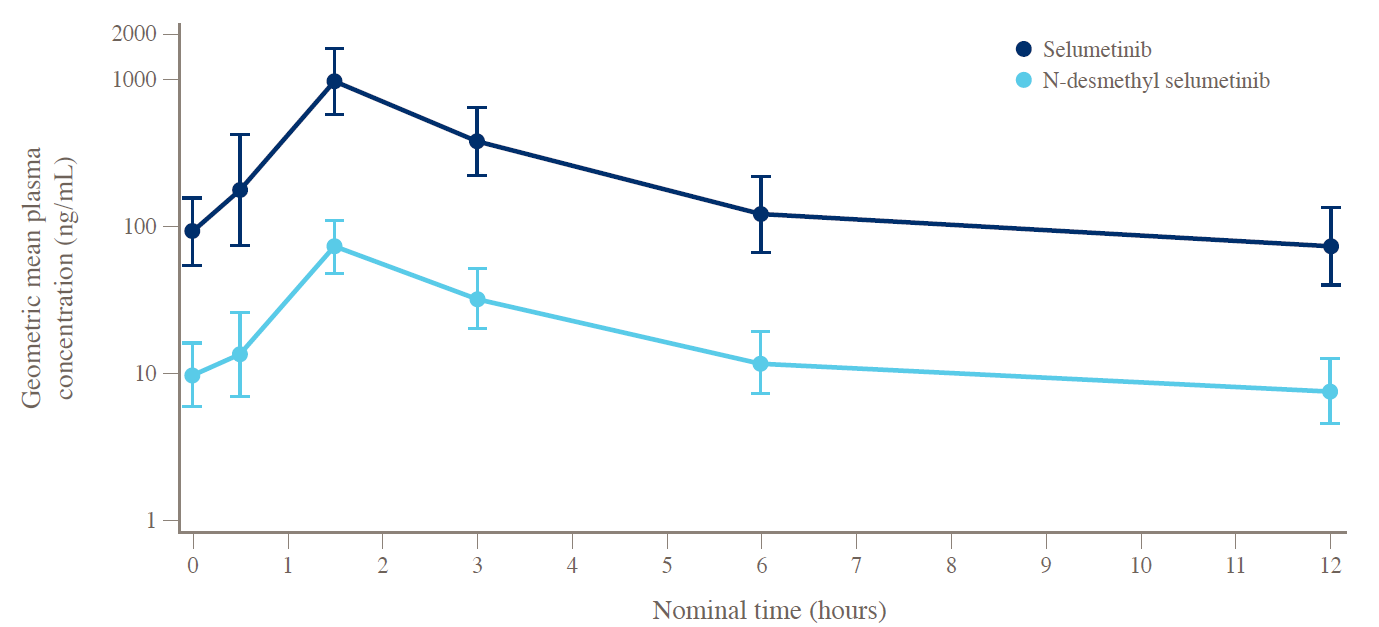


Vertical lines represent ×/÷ geometric standard deviation. Gmean × gSD = EXP (AriMean in log scale + SD in log scale). Gmean ÷ gSD = EXP (AriMean in log scale - SD in log scale).
AriMean, arithmetic mean; EXP, exponential; Gmean, geometric mean; gSD, geometric standard deviation; log, logarithmic; PK, pharmacokinetics; SD, standard deviation.

FIGURE S2 Best percentage change from baseline in target PN volume in adult patients per (A) ICR and (B) investigator and pediatric patients per (C) ICR and (D) investigator.

**A
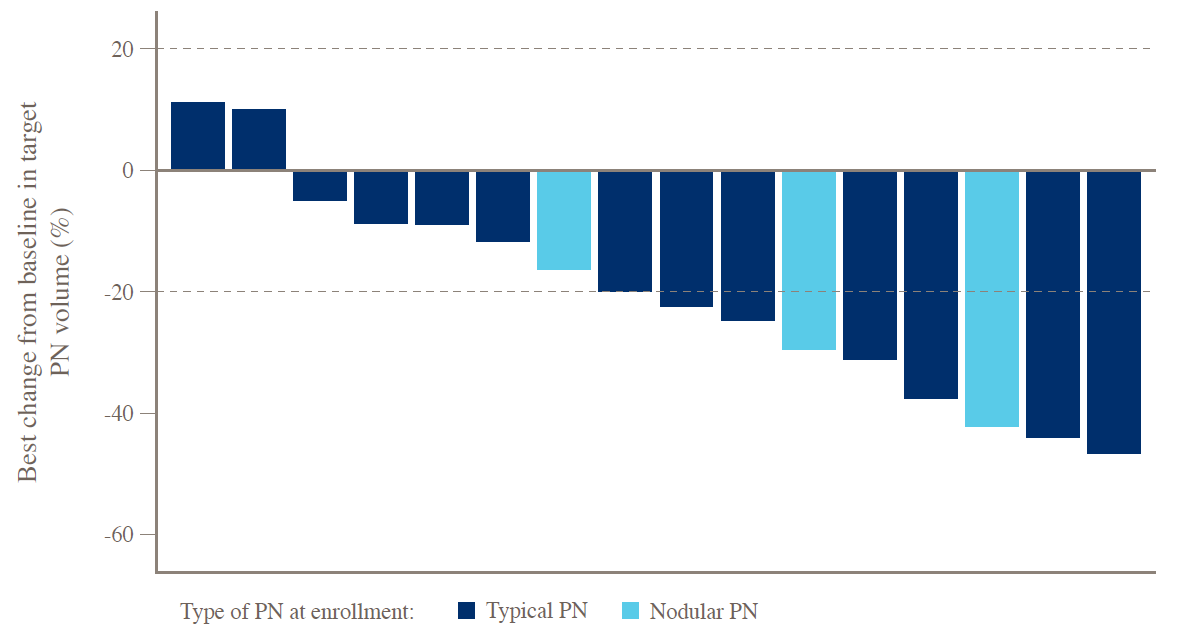
**

**B
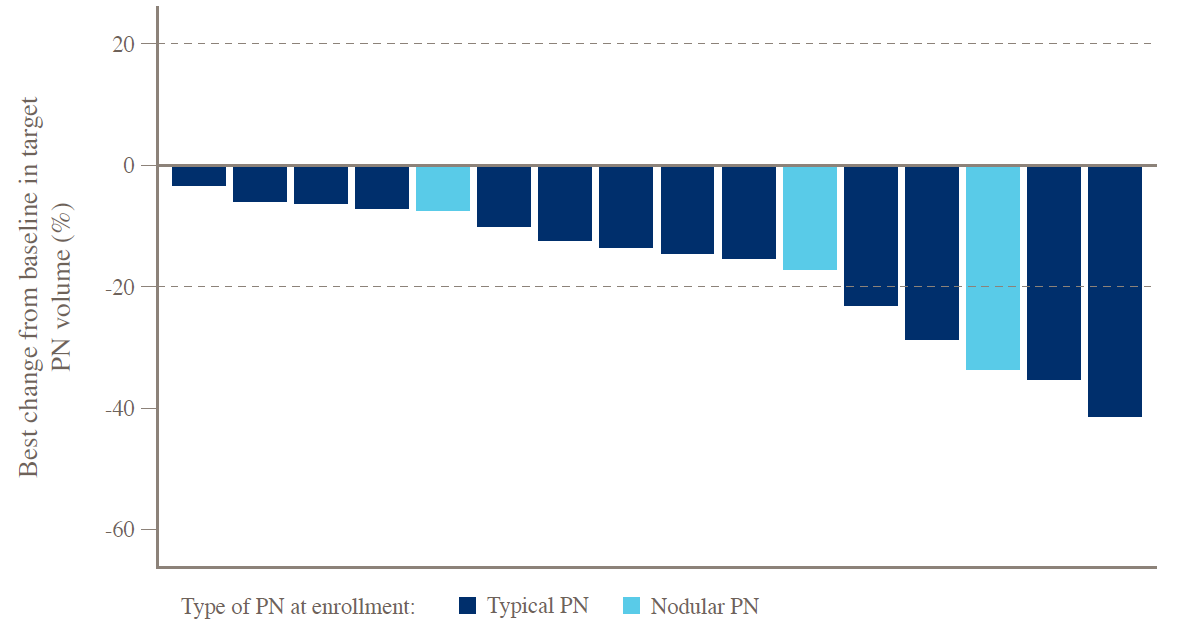
**

**C**


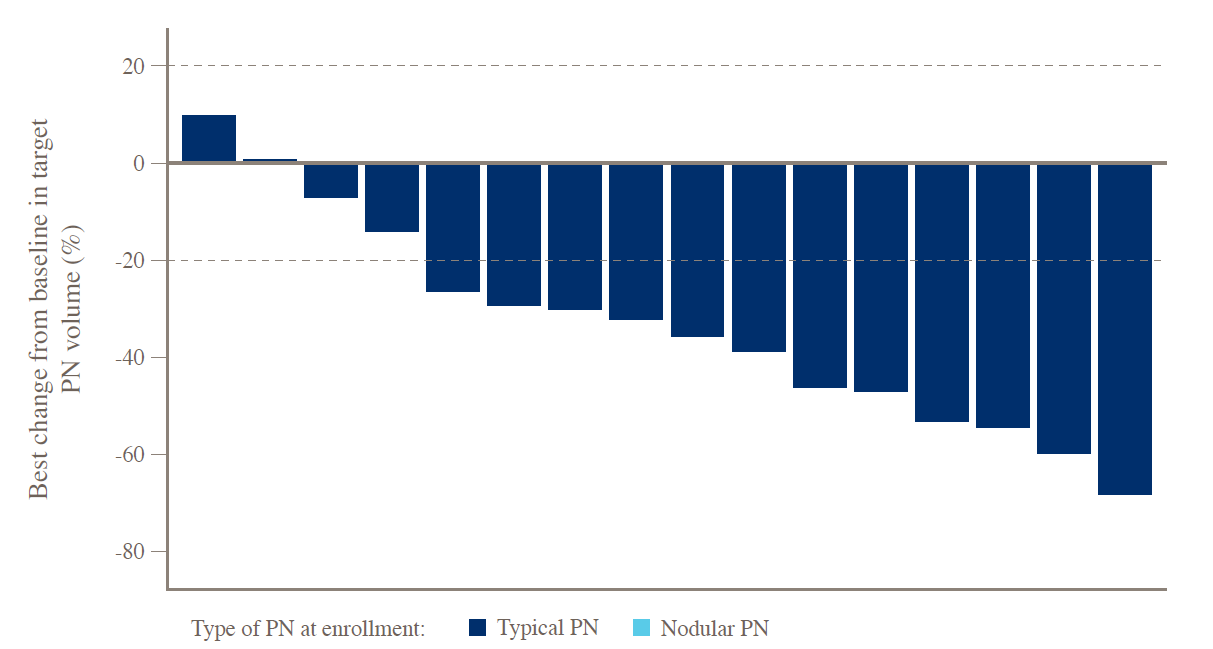


**D**

**
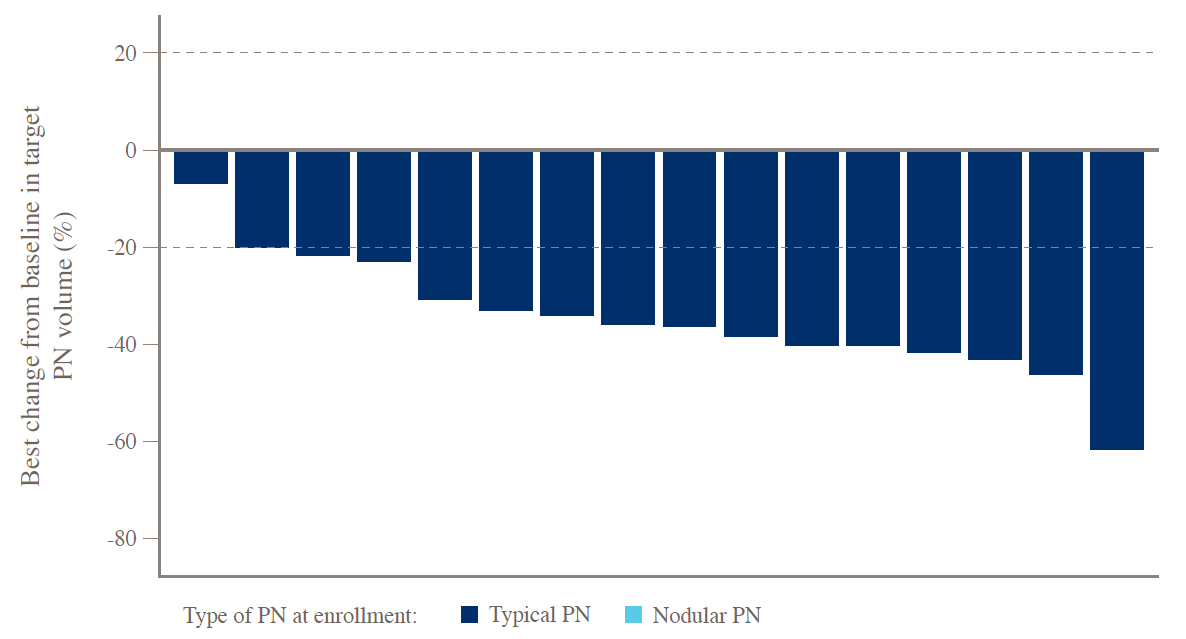
**

Each bar represents one patient’s best percentage change in target PN volume. Bars are ordered from the largest increase to the largest decrease of PN volume assessed by ICR or investigator. The order of the bars per ICR and investigator assessment do not correspond. Best percentage change was derived as the maximum reduction from baseline or the minimum increase from baseline to data cut-off in the absence of reduction. ICR, independent central review; PN, plexiform neurofibroma.

FIGURE S3 Percentage change in target PN volume over time from baseline according to REiNS criteria for adult patients (A investigator assessment and B ICR assessment) and pediatric patients (C investigator assessment and D ICR assessment).

**A**


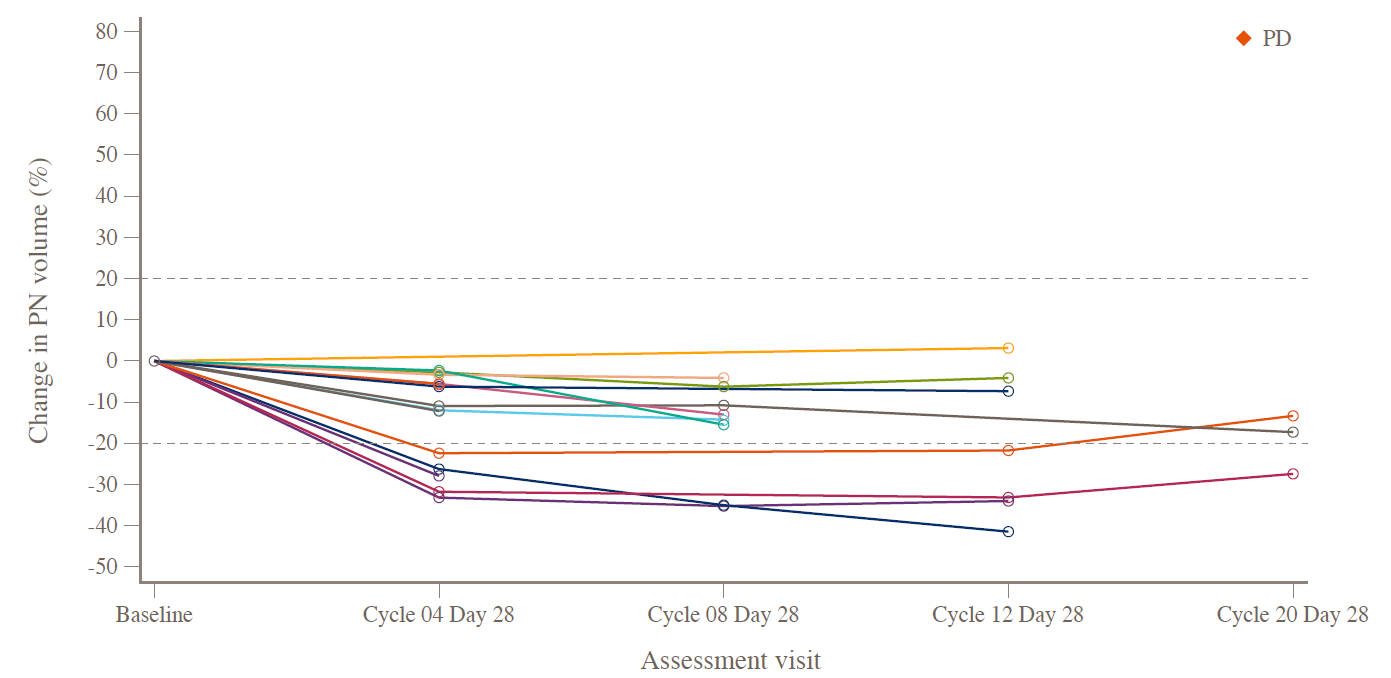


**B**


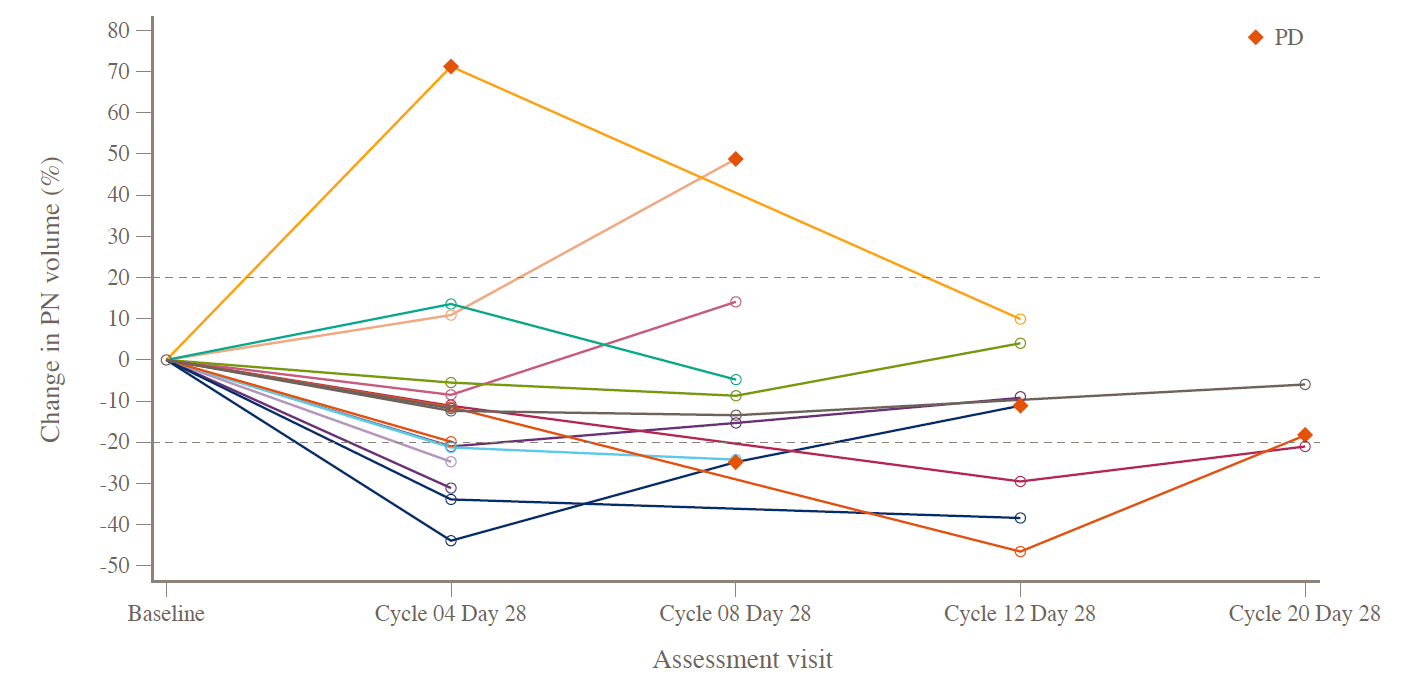


**C**


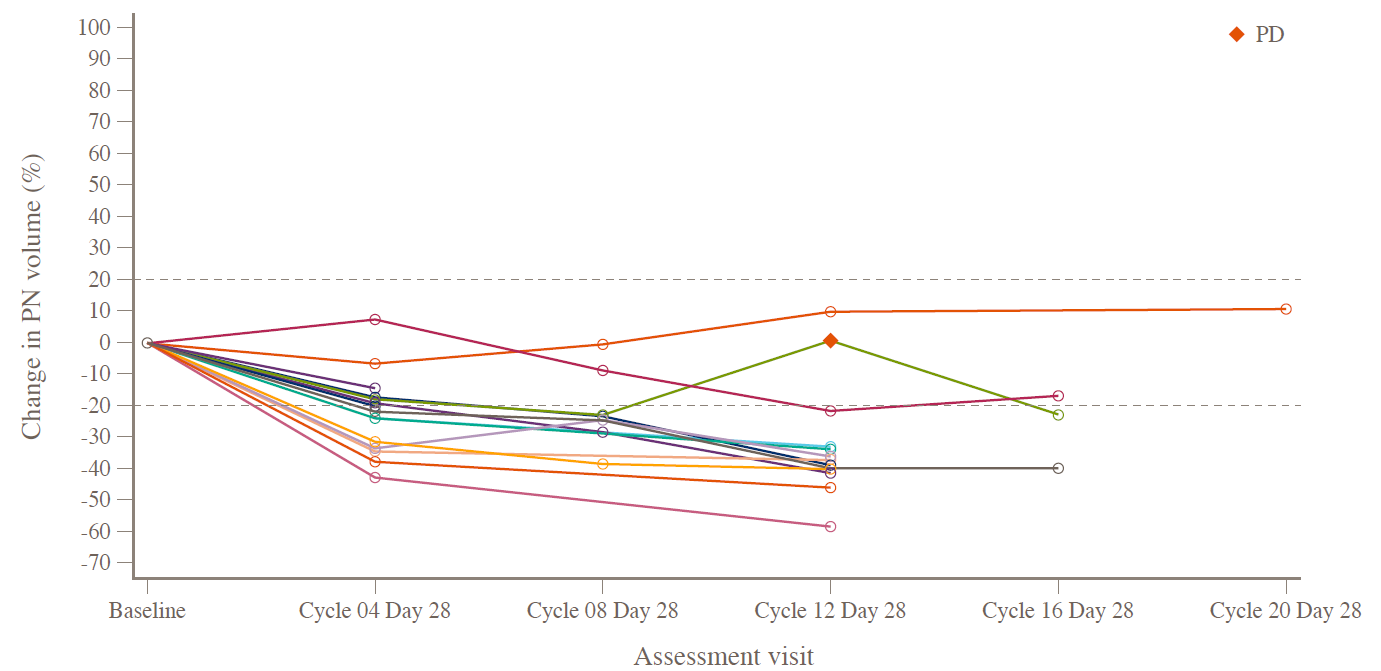


**D**


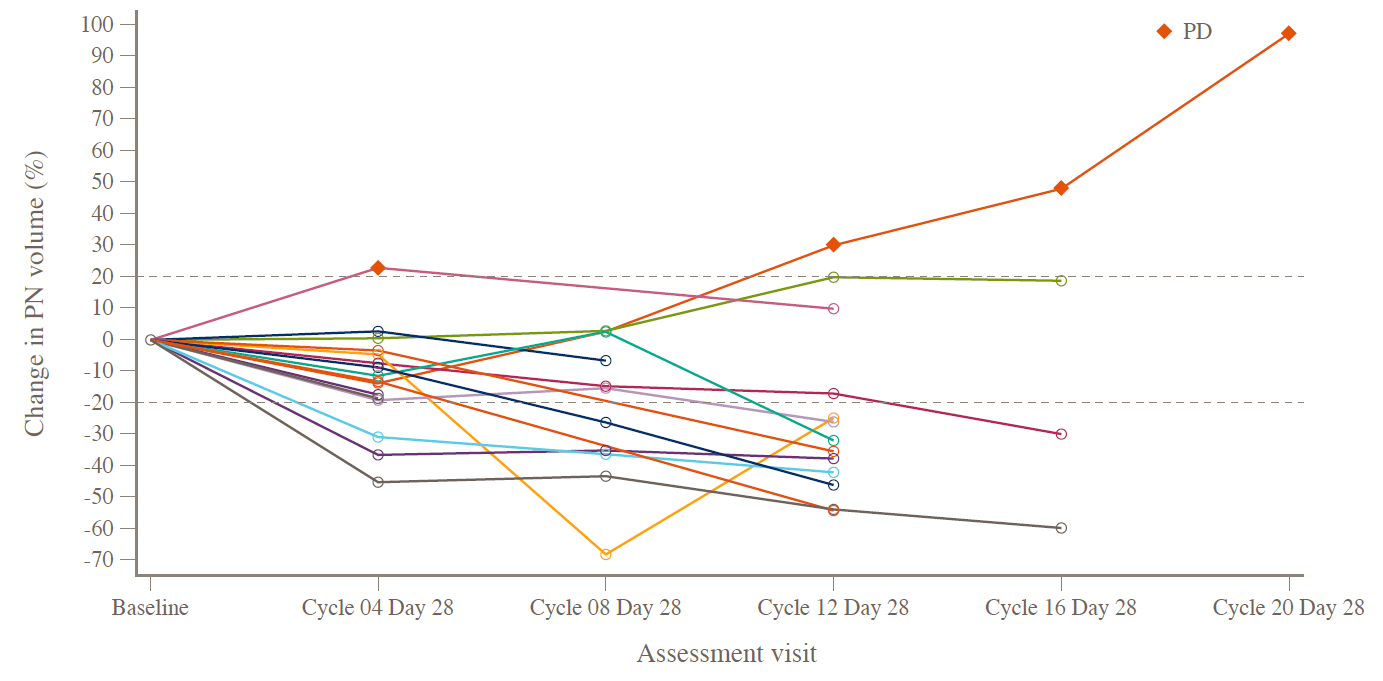


PD was defined as a 20% or more increase in target PN volume relative to baseline or the best overall response (maximum tumor reduction) recorded after PR. The reference line represents a 20% change in tumor volume.
ICR, independent central review; PD, progressive disease; PN, plexiform neurofibroma; PR, partial response; REiNS, Response Evaluation in Neurofibromatosis and Schwannomatosis.

FIGURE S4 Best percentage change in non-target PN volume changes from baseline according to REiNS criteria for adult patients (A investigator assessment and B ICR assessment) and pediatric patients (C investigator assessment and D ICR assessment).

**A**

**
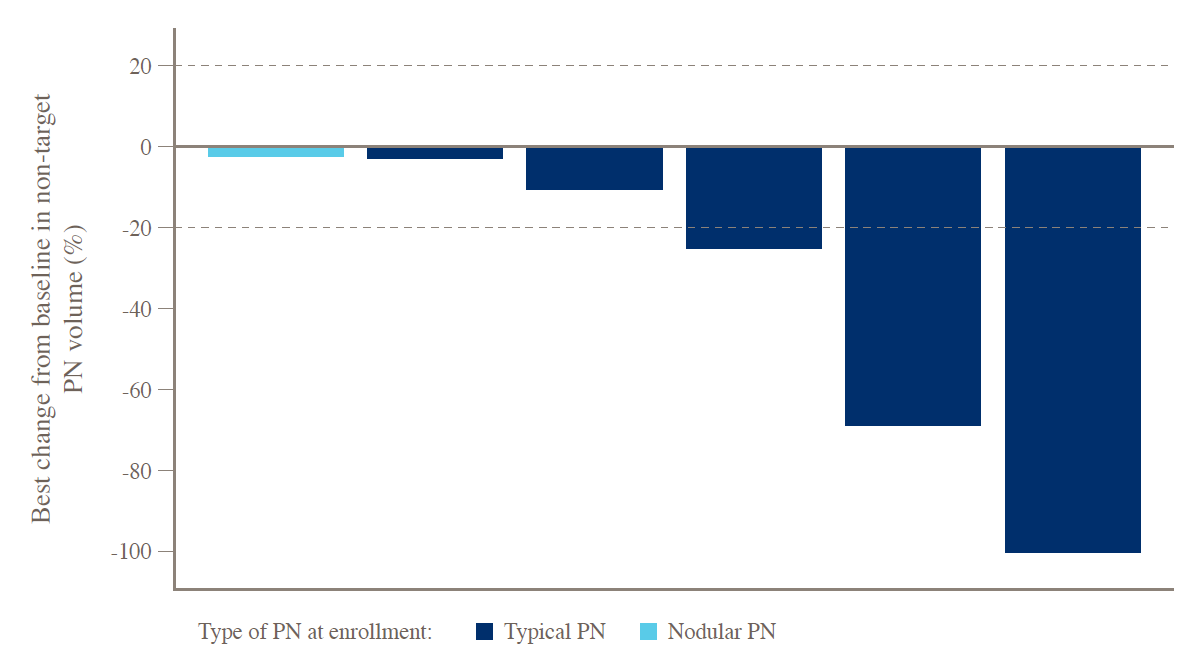
**

**B**

**
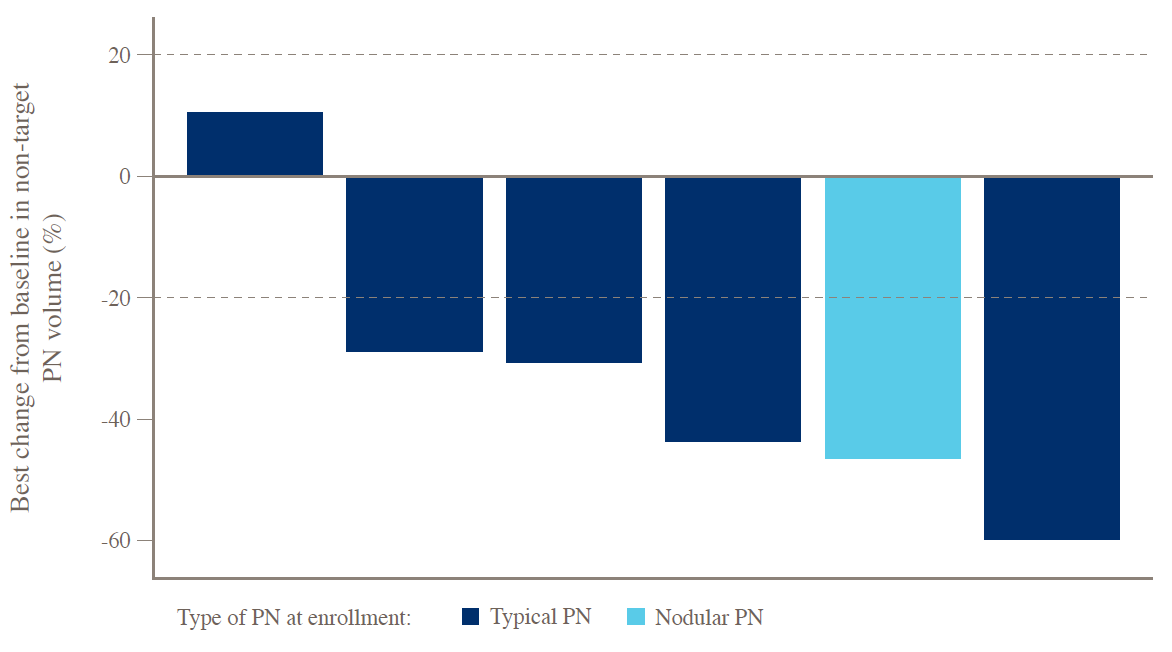
**

**C**


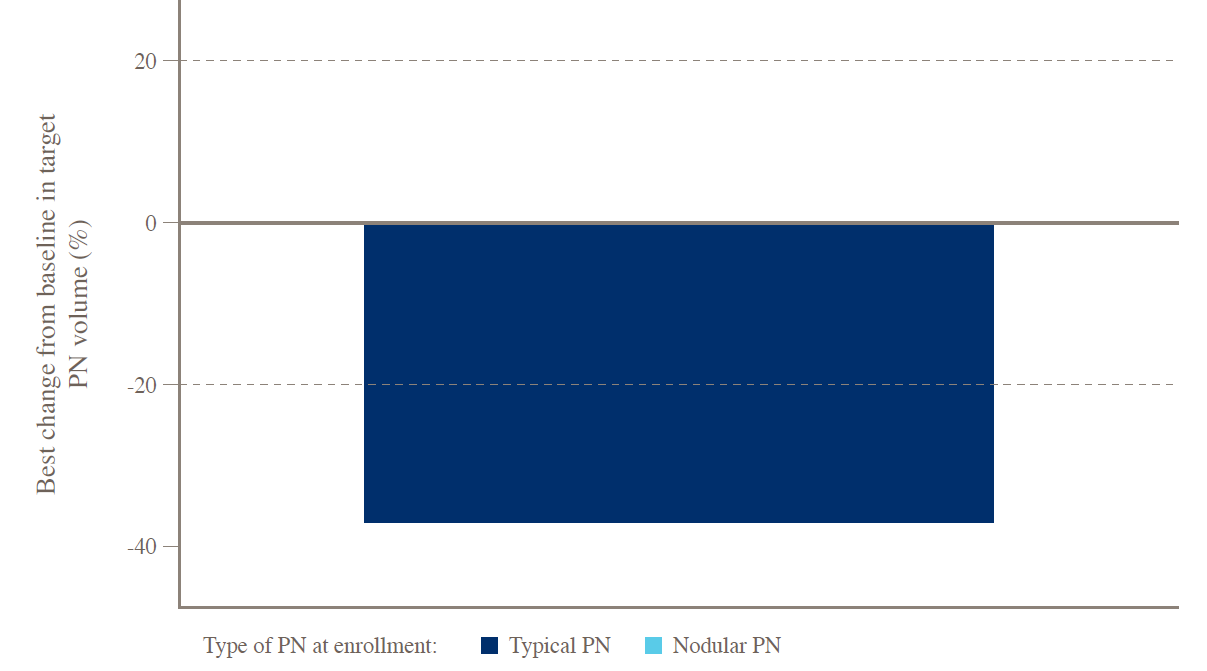


**D**

**
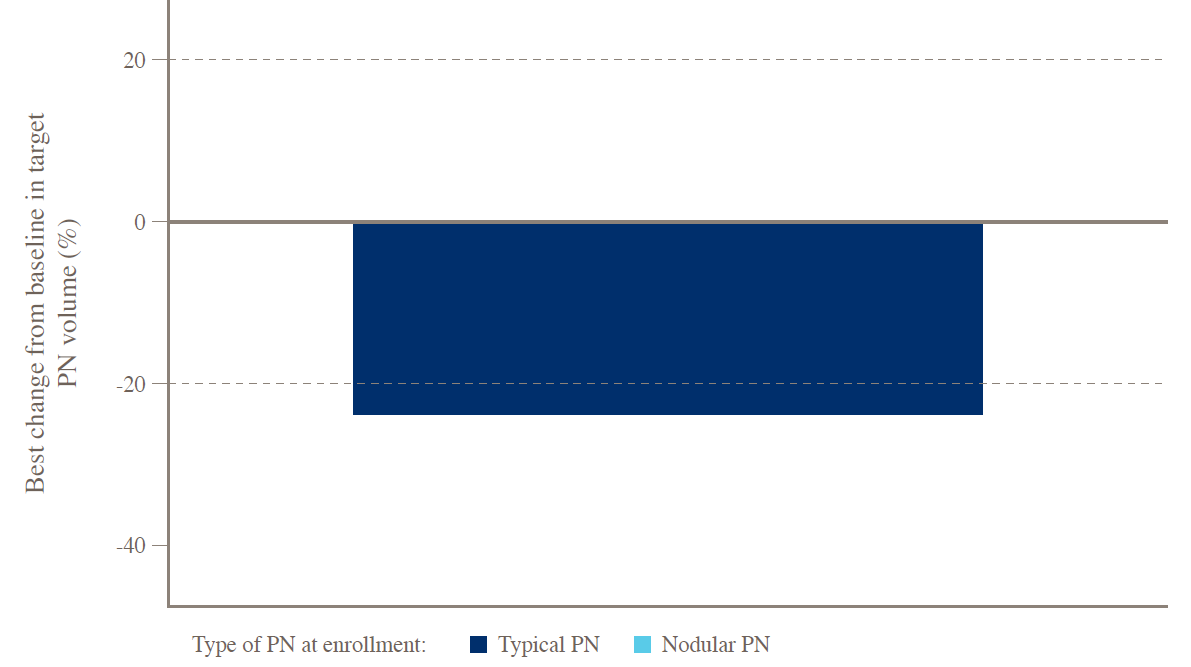
**

Each bar represents one patient’s best percentage change in non-target PN volume. The bars are ordered from the largest increase to the largest decrease of PN volume assessed by investigator or ICR. The order of the bars of investigator and ICR assessment do not correspond. Best percentage change was derived as the maximum reduction from baseline or the minimum increase from baseline to data cut-off in the absence of reduction. A negative change denotes a reduction in target lesion size. ICR, independent central review; PN, plexiform neurofibroma; REiNS, Response Evaluation in Neurofibromatosis and Schwannomatosis.

FIGURE S5 Adjusted mean pain intensity change from baseline per NRS-11 (physician-selected tumor pain) by MMRM analysis (safety analysis set) for (A) adult patients and (B) pediatric patients per Faces pain scale by MMRM analysis (safety analysis set).

**A**


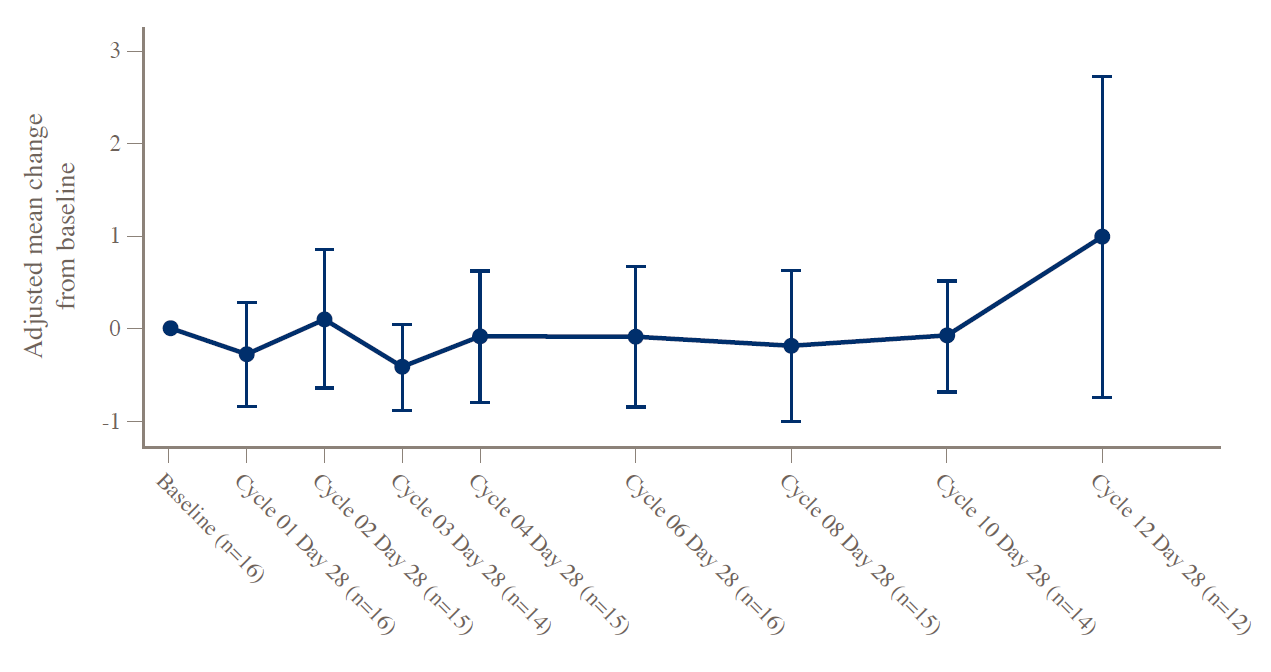


**B**


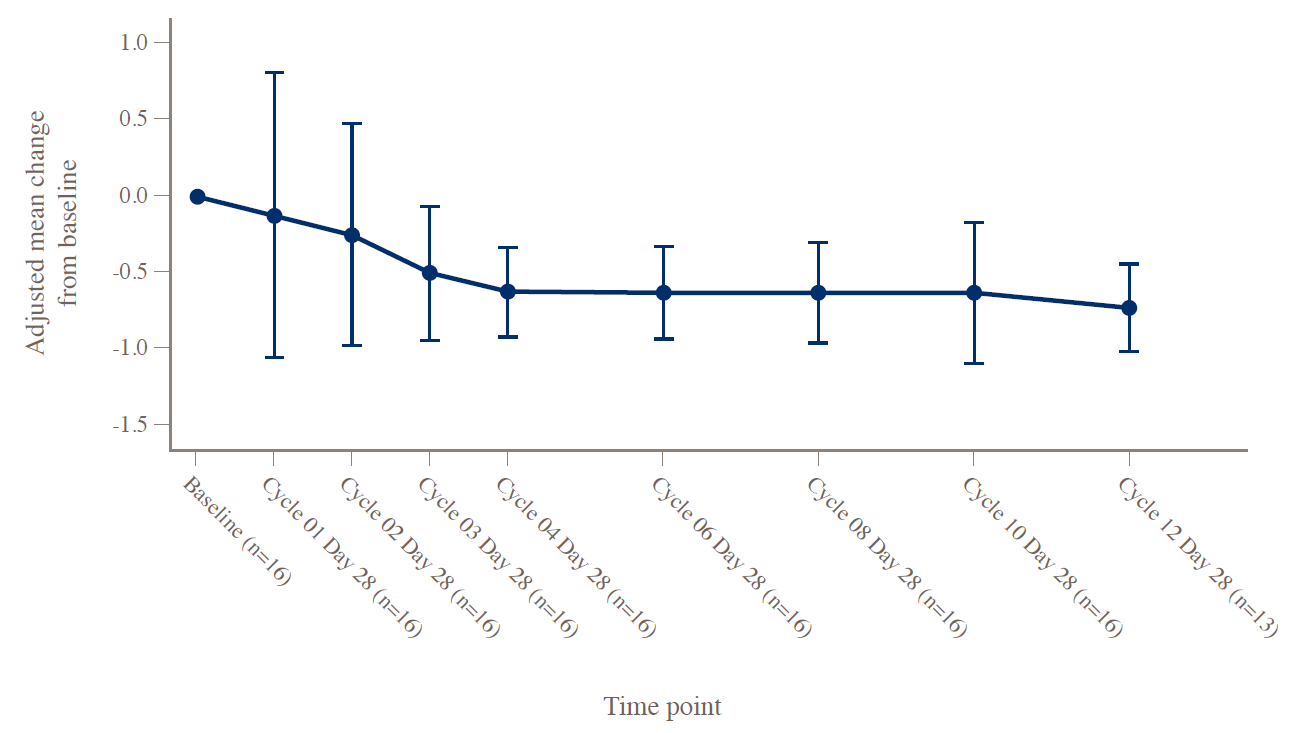


Children (4–17 years) at enrolment completed Faces pain scale. A higher score represents more pain. Error bars represent 95% confidence intervals for each respective adjusted mean change from baseline. The analysis was performed using a MMRM analysis of change from baseline for all post-baseline visits, with baseline score in the model as a covariate and scheduled visit as an explanatory variable. Only visits with at least 10 patients were included. Baseline was defined as the last result obtained prior to the start of study treatment.

MMRM; mixed model repeated measures; NRS-11, numerical rating scale-11.

FIGURE S6 Adjusted PII total scores change from baseline over time by MMRM analysis (safety analysis set) in the (A) adult and pediatric (B self-reported, C caregiver-reported).

**A**


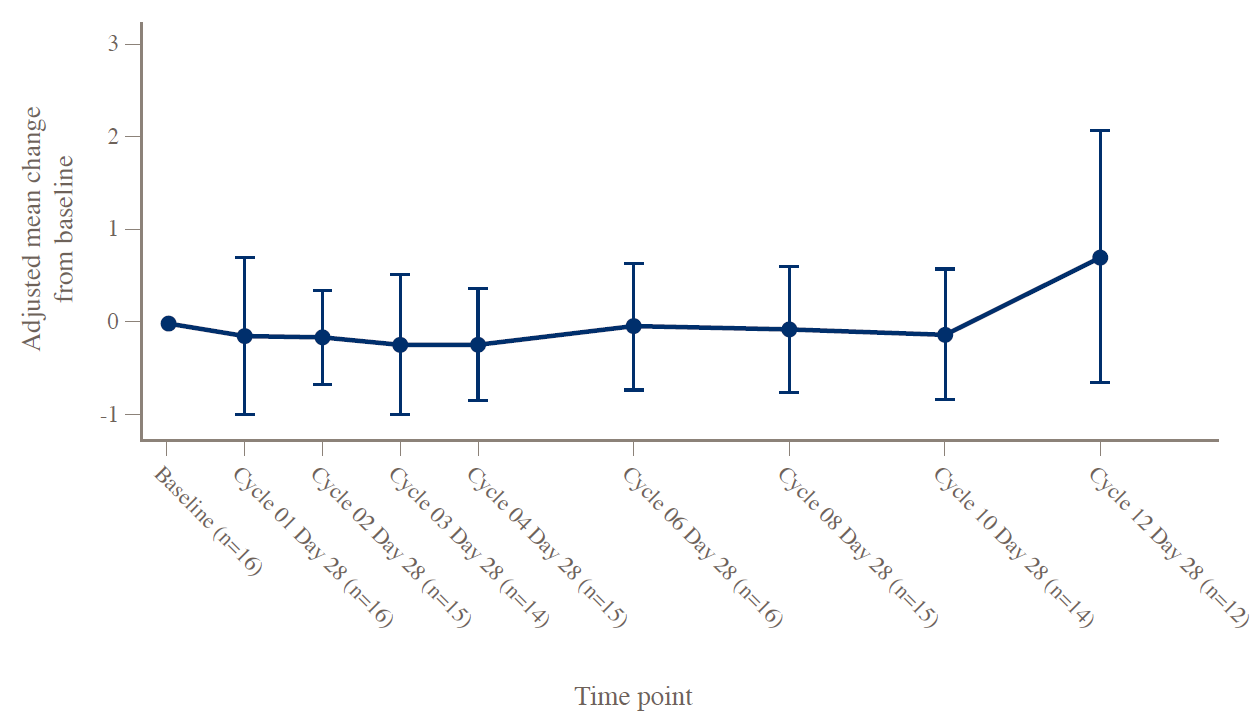


**B**


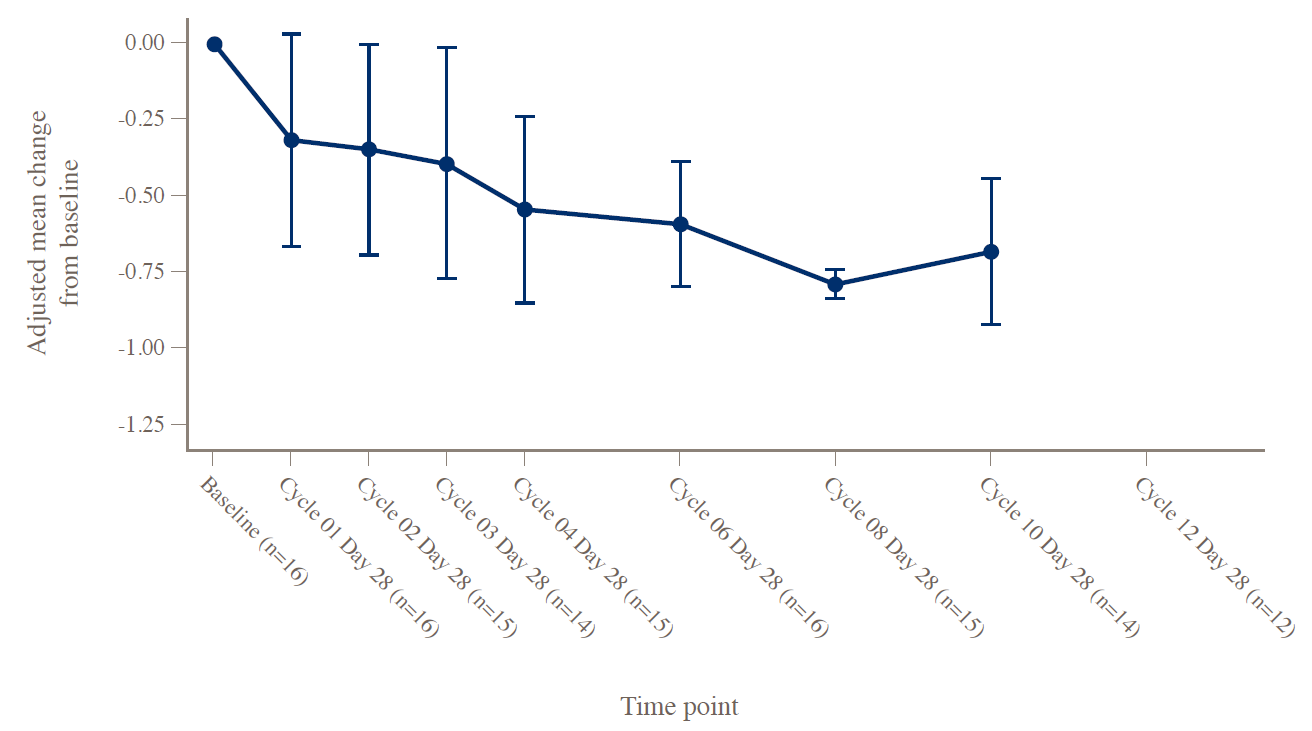


**C**


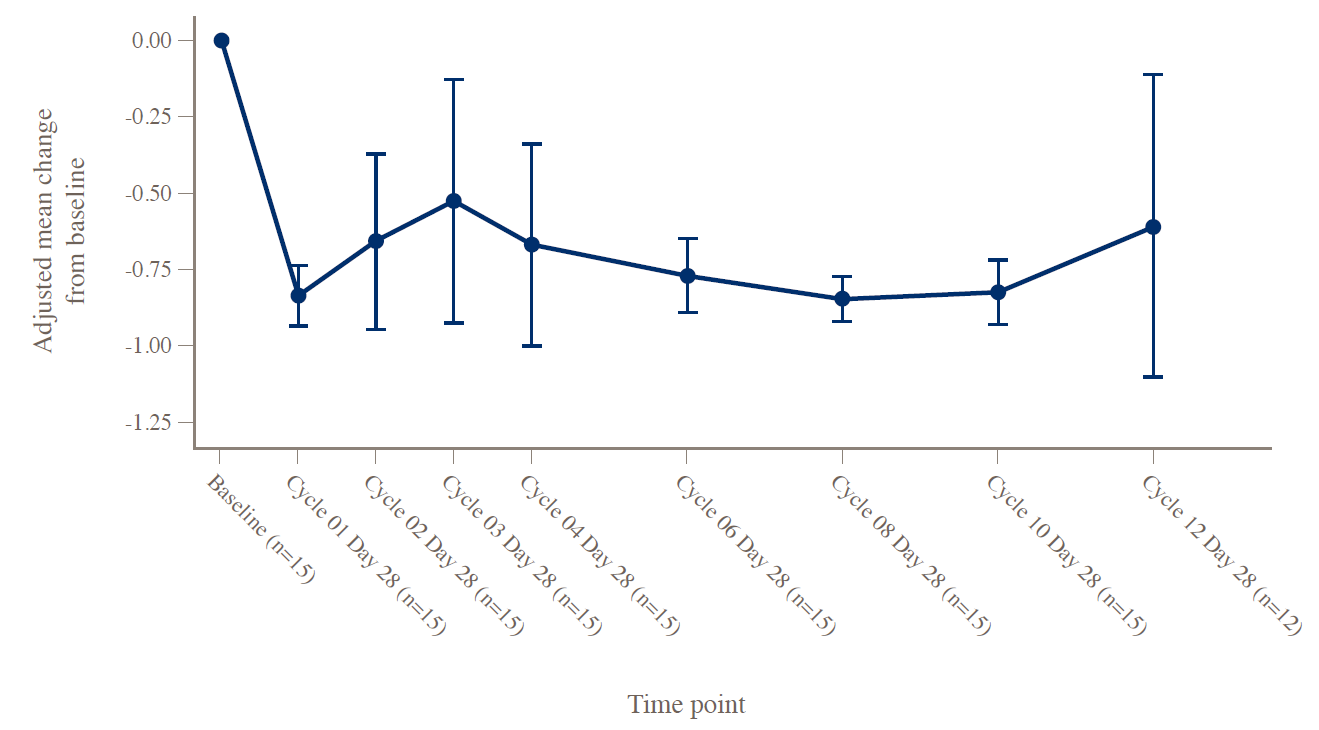


Adults and children (8–17 years) completed self-reported PII, and caregiver-reported PII was reported for children 5–17 years old. A higher score represents more interferences on daily activities. Error bars represent 95% confidence intervals for each respective adjusted mean change from baseline. The analysis was performed using a MMRM analysis of change from baseline for all post-baseline visits, with baseline score in the model as a covariate and scheduled visit as an explanatory variable. Only visits with more than 10 patients were included. Baseline was defined as the last result obtained prior to the start of study treatment.

MMRM, mixed model repeated measures; PII, pain interference index.

FIGURE S7 Adjusted mean self-/caregiver-reported PROMIS scores change from baseline over time by MMRM analysis (safety analysis set) in the adult (A short form) and pediatric (Mobility: B self-reported, C caregiver-reported. Upper extremity: D self-reported, E caregiver-reported) cohorts.

**A**
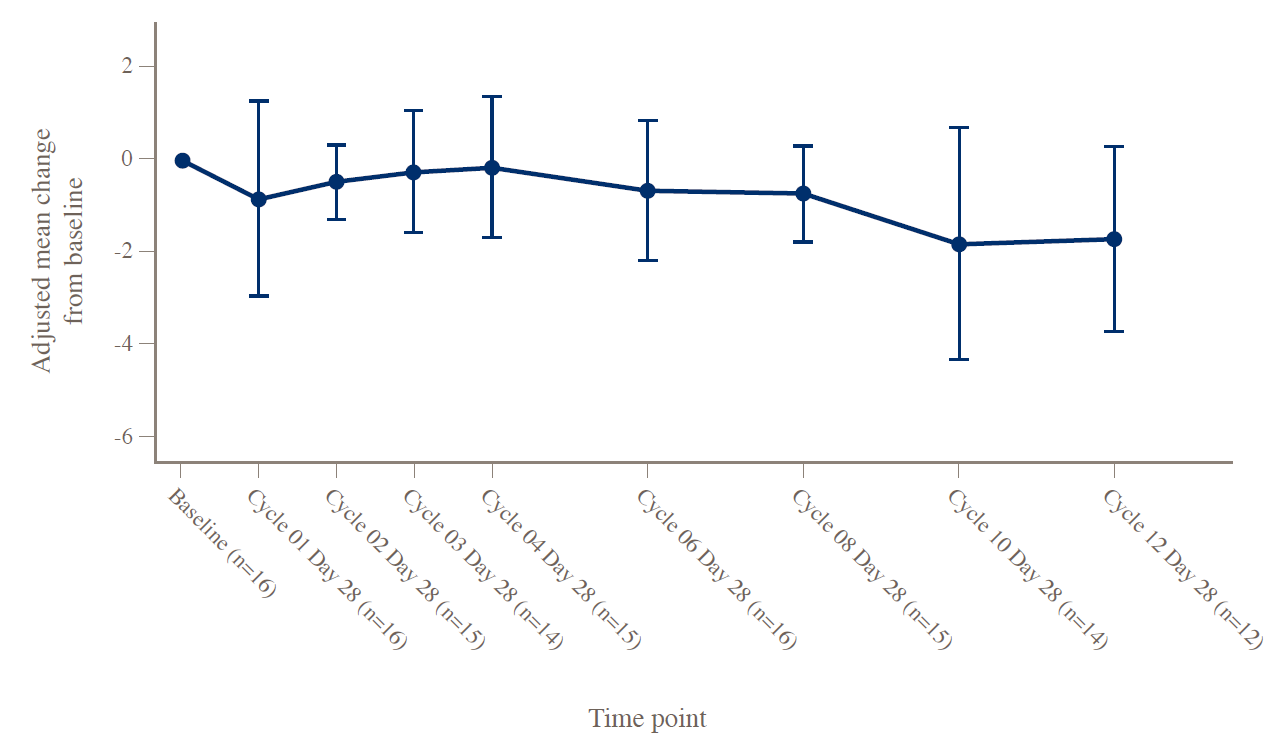


**B**


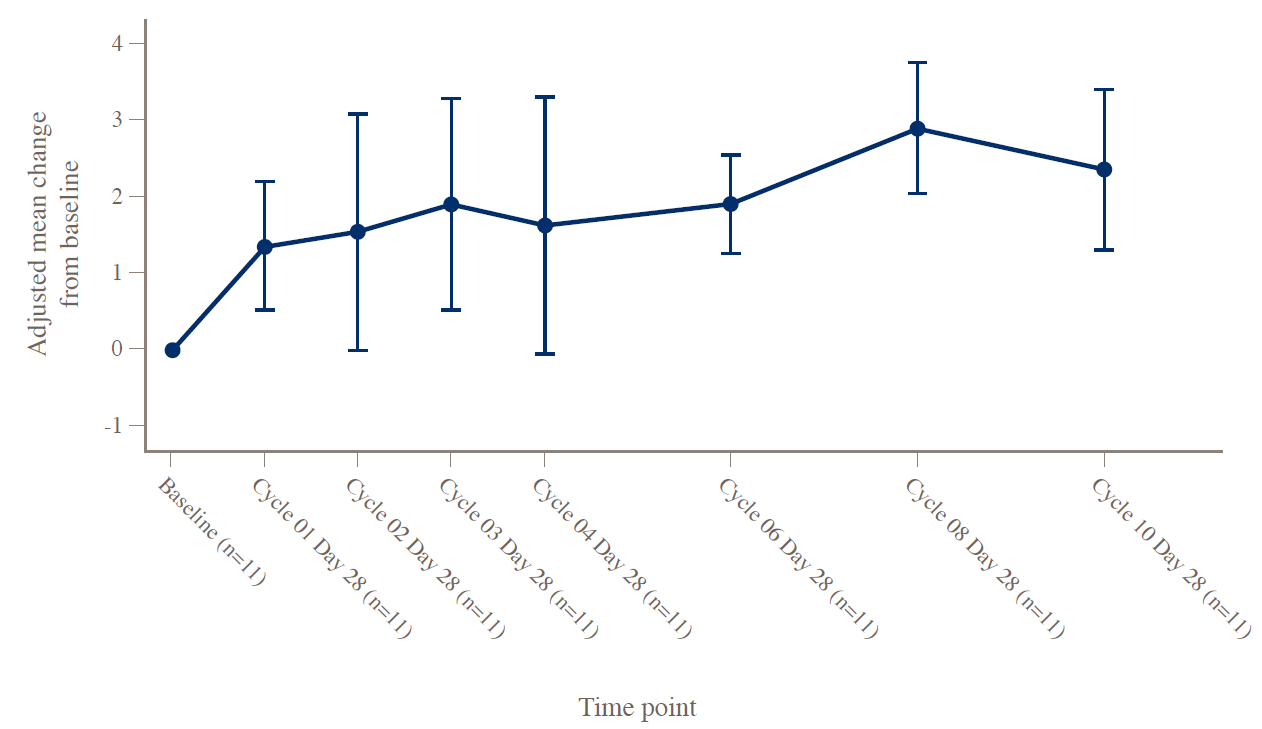


**C**


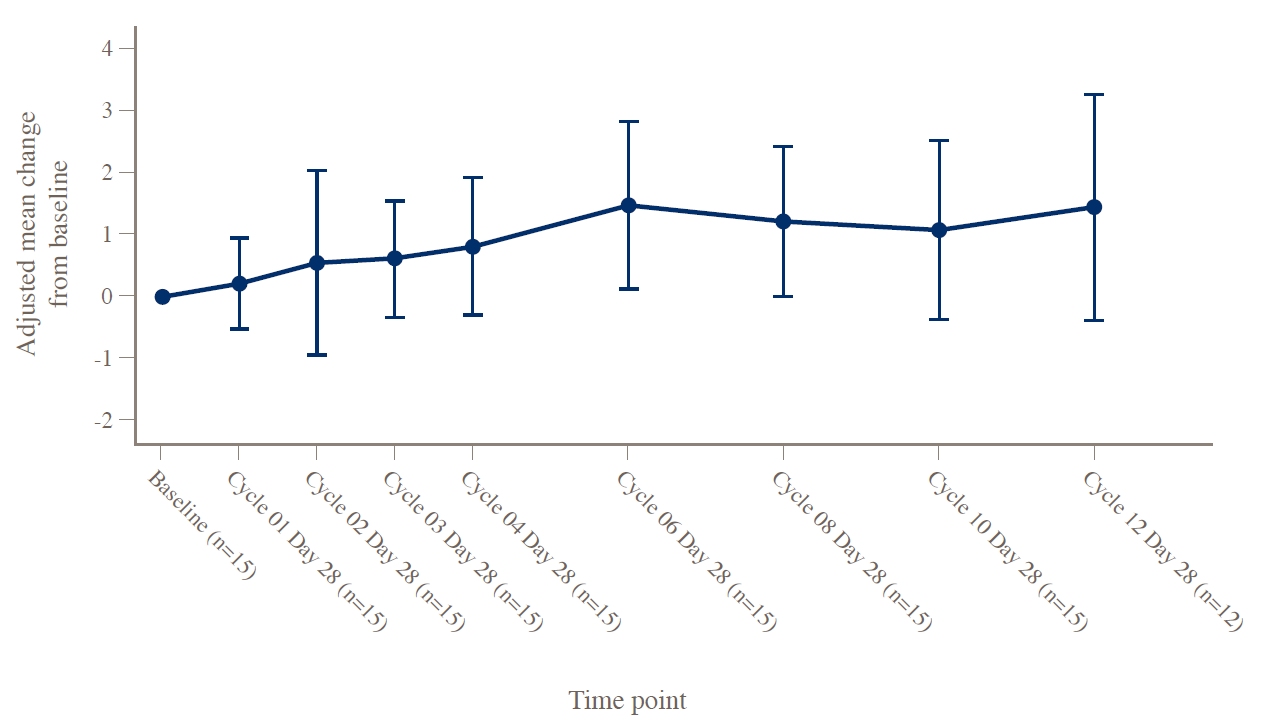


**D**


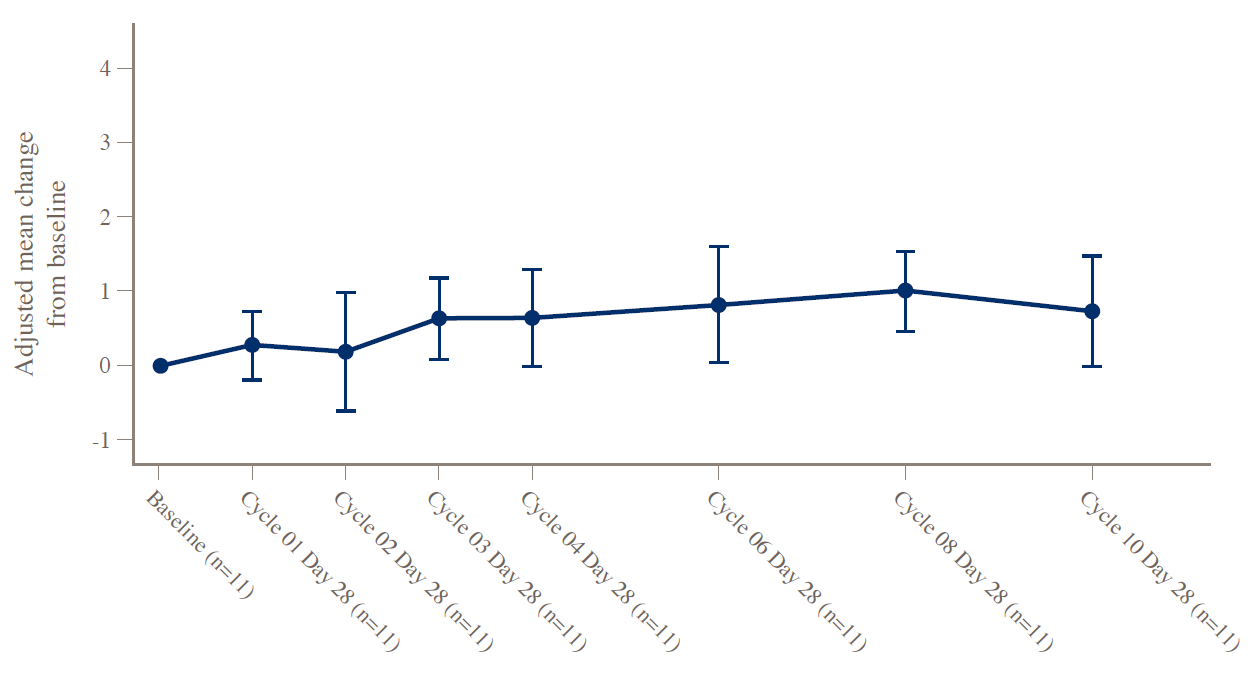


**E**


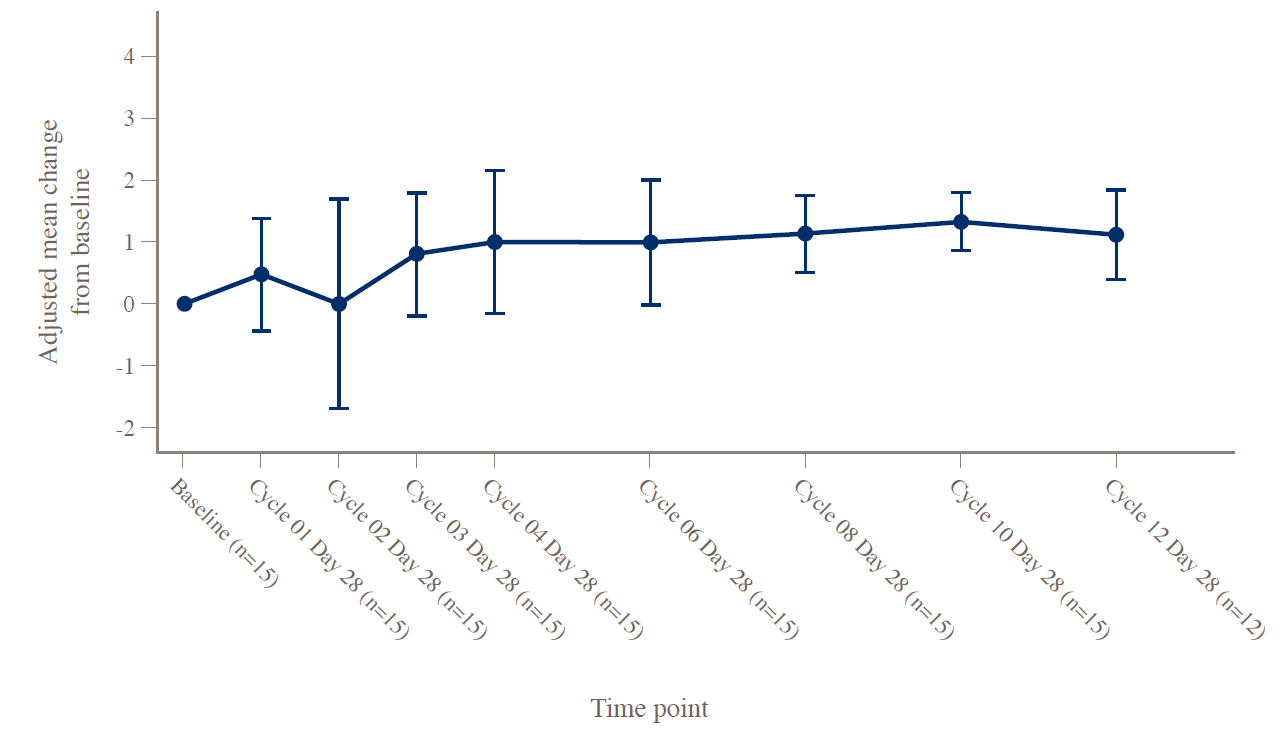


Patients aged 8–17 years completed the self-reported questionnaire. The caregiver-reported questionnaire was completed for patients aged 5–17 years. Higher scores indicate better physical functioning. The analysis was performed using an MMRM analysis of change from baseline for all post-baseline visits, with baseline score in the model as a covariate and schedules visit as an exploratory variable. Only visits with more than 10 patients were included. Baseline was defined as the last result obtained before the start of study treatment. MMRM, mixed model repeated measures; PROMIS, Patient-Reported Outcomes Measurement Information System.

FIGURE S8 (A) Adjusted mean PlexiQoL scores change from baseline by MMRM analysis for the adult cohort and the adjusted mean PedsQL self- and caregiver-reported total scores, change from baseline over time by MMRM analysis for the pediatric cohort (B self-reported, C caregiver-reported).

**A**


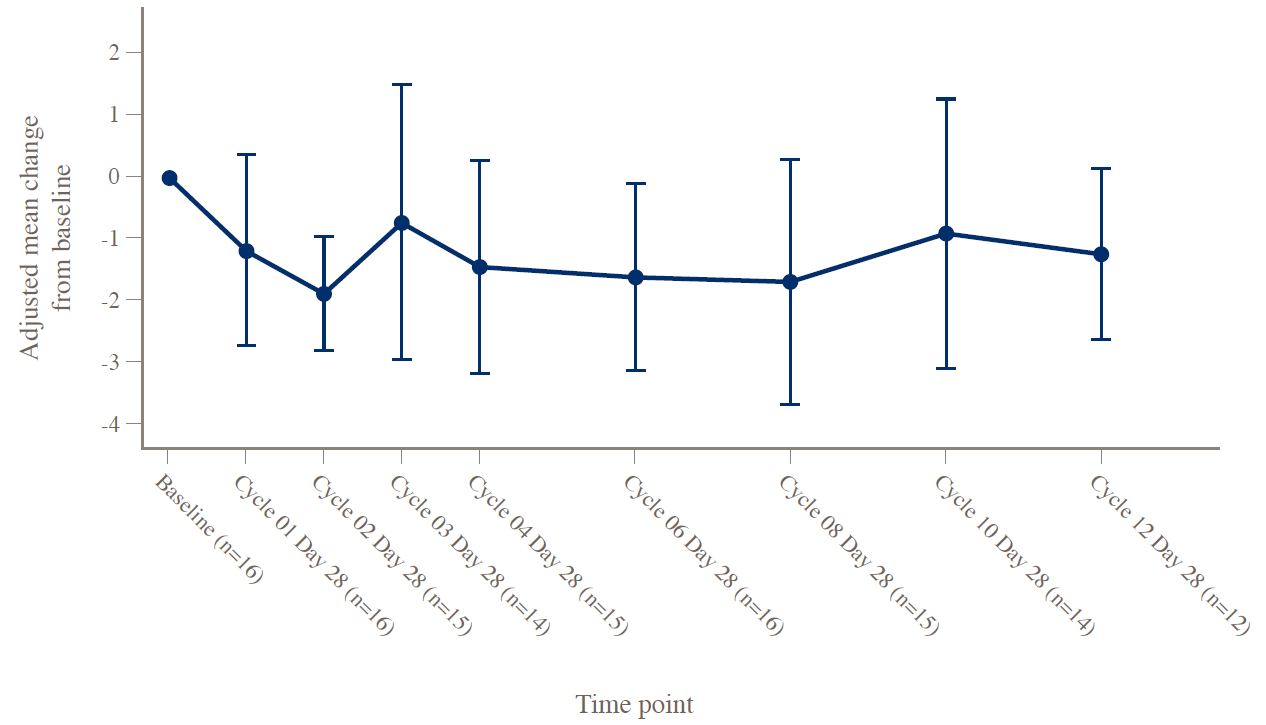


**B**


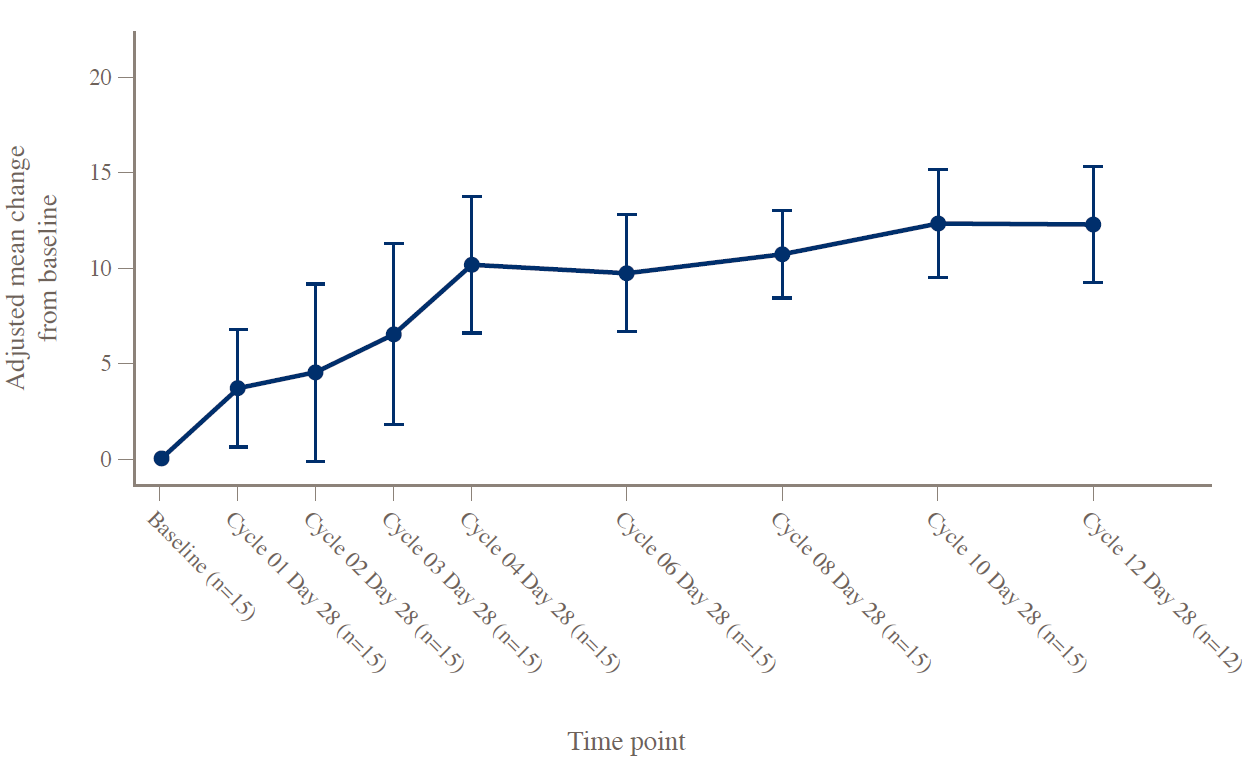


**C**


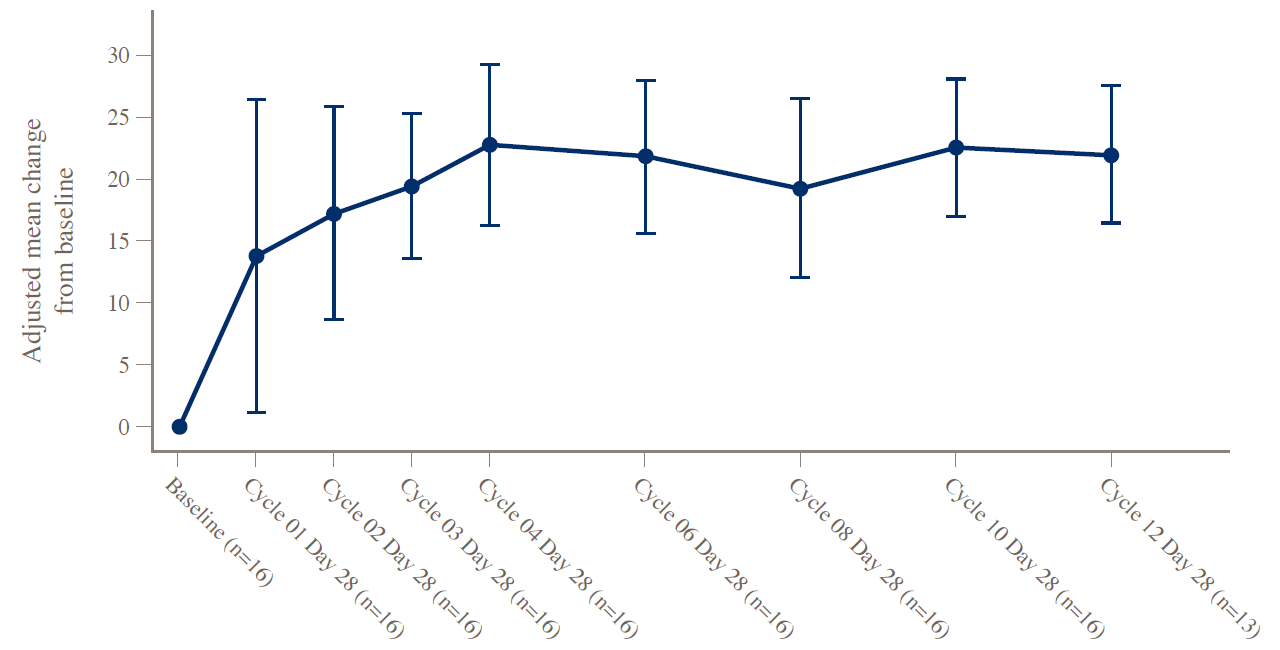


A lower PlexiQoL score is favorable. Patients aged 5–17 years completed the self-report questionnaire, and the caregiver-reported questionnaire was completed for all pediatric patients. A higher PedsQL score is favorable. Error bars represent 95% confidence intervals for each respective adjusted mean change from baseline. The analysis was performed using a MMRM analysis of change from baseline for all post-baseline visits, with baseline score in the model as a covariate and schedules visit as an exploratory variable. Only visits with more than 10 patients were included. Baseline was defined as the last result obtained prior to the start of study treatment. MMRM, mixed model repeated measures; PedsQL, Pediatric Quality of Life Inventory; PlexiQoL, plexiform neurofibromas quality of life scale.

FIGURE S9 Patient’s global impression of change over time for (A) adult patients (self-reported) and pediatric (B self-reported, C caregiver-reported) over time compared with baseline (safety analysis set).

**A
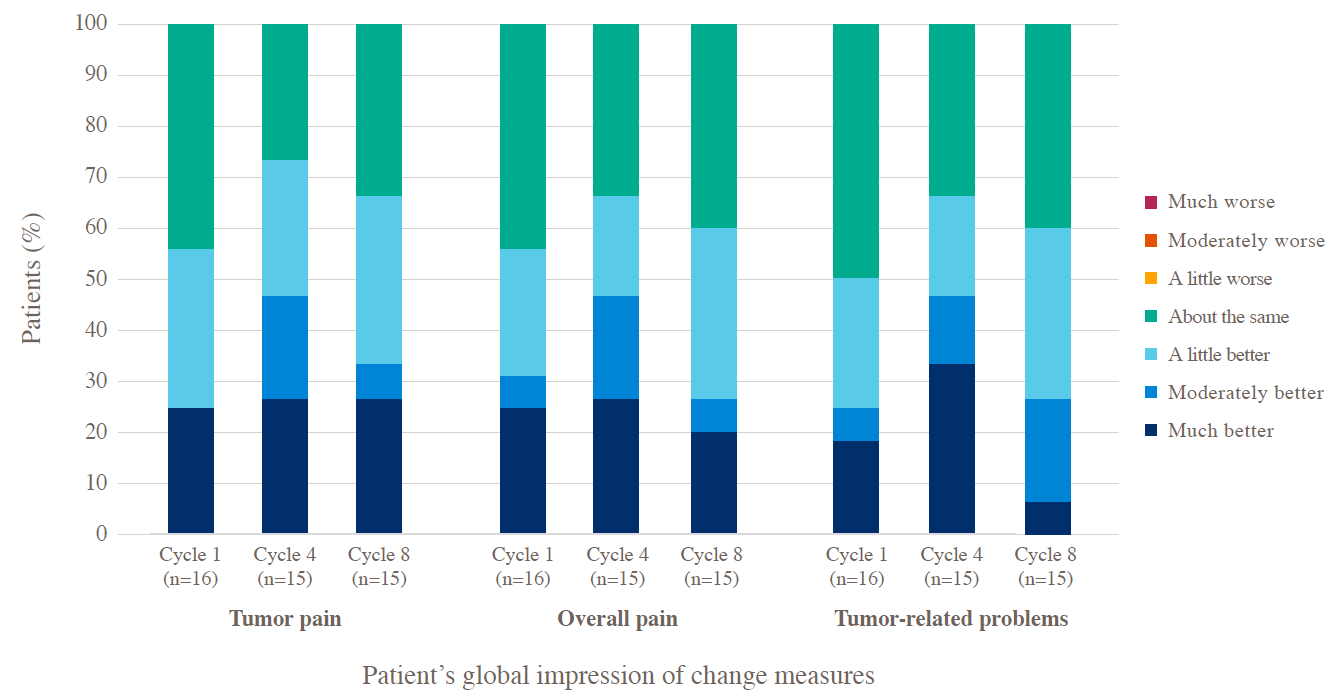
**

**B
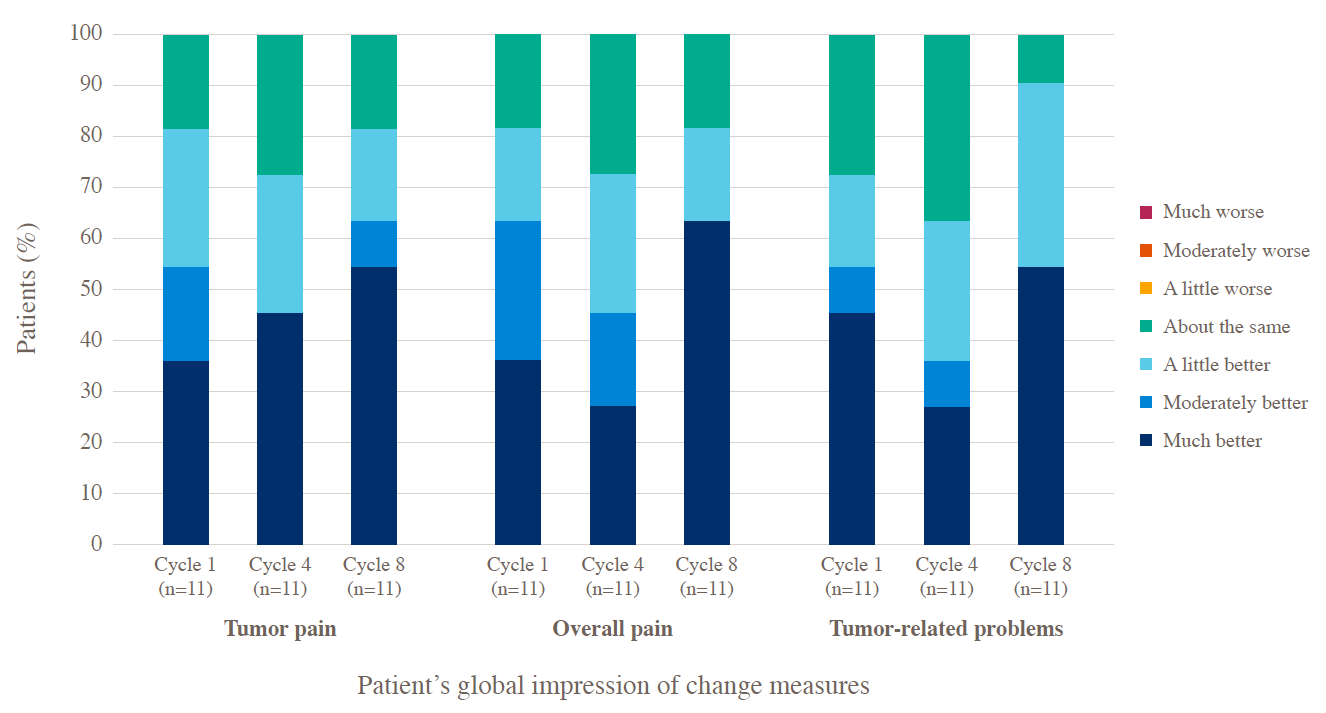
**

**C
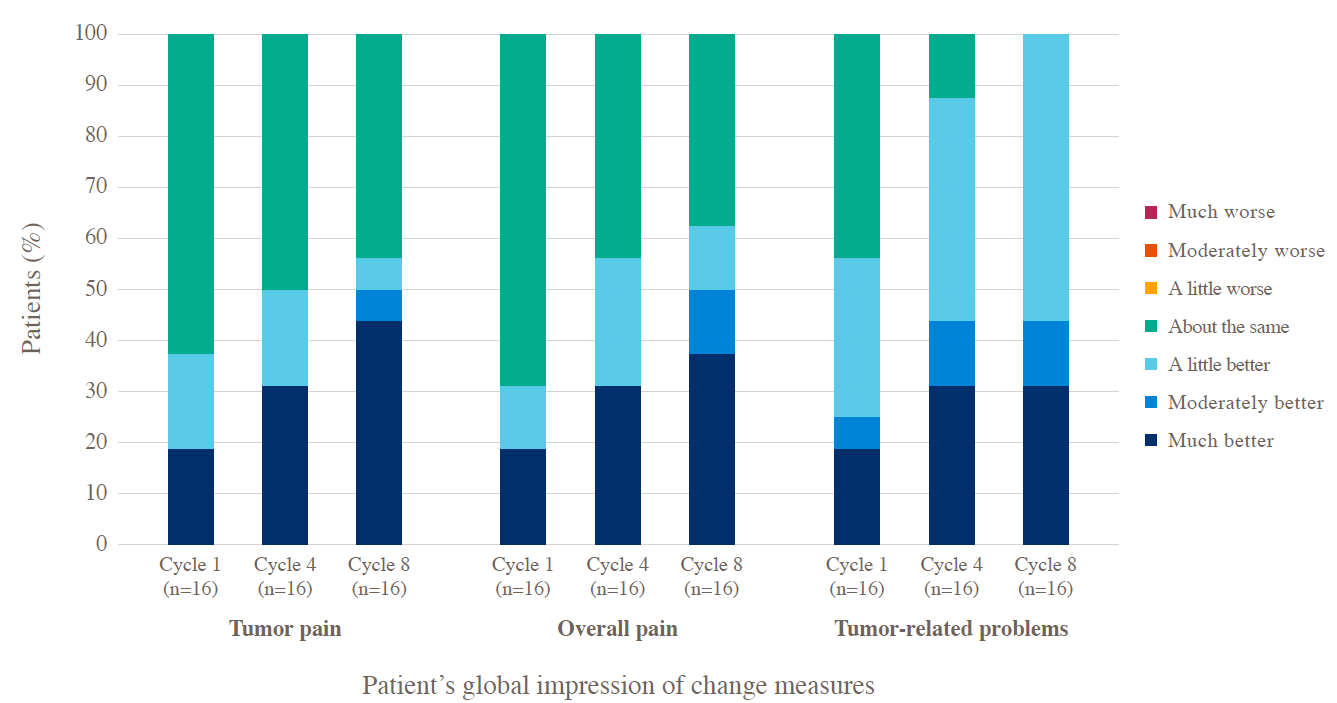
**

FIGURE S10 Patient’s global impression of severity over time for (A) adult patients (self-reported) and pediatric (B self-reported, C caregiver-reported) and over time (safety analysis set).

**A
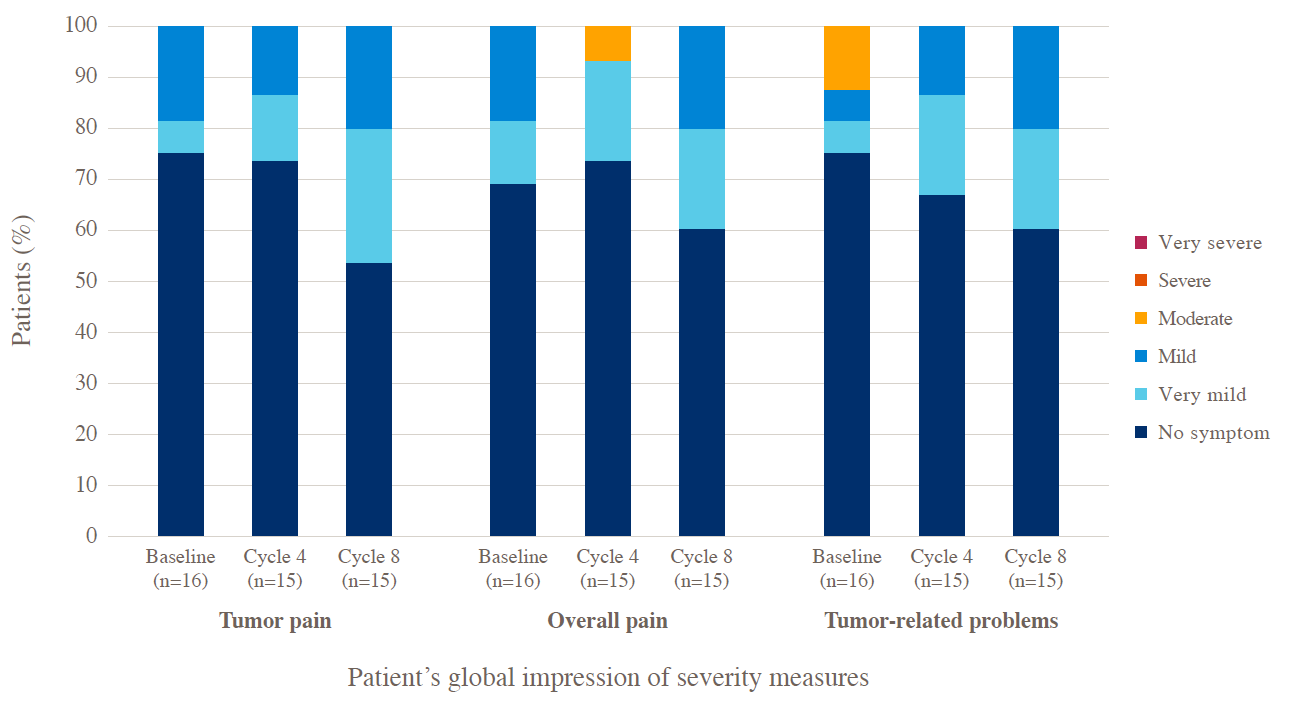
**

**B
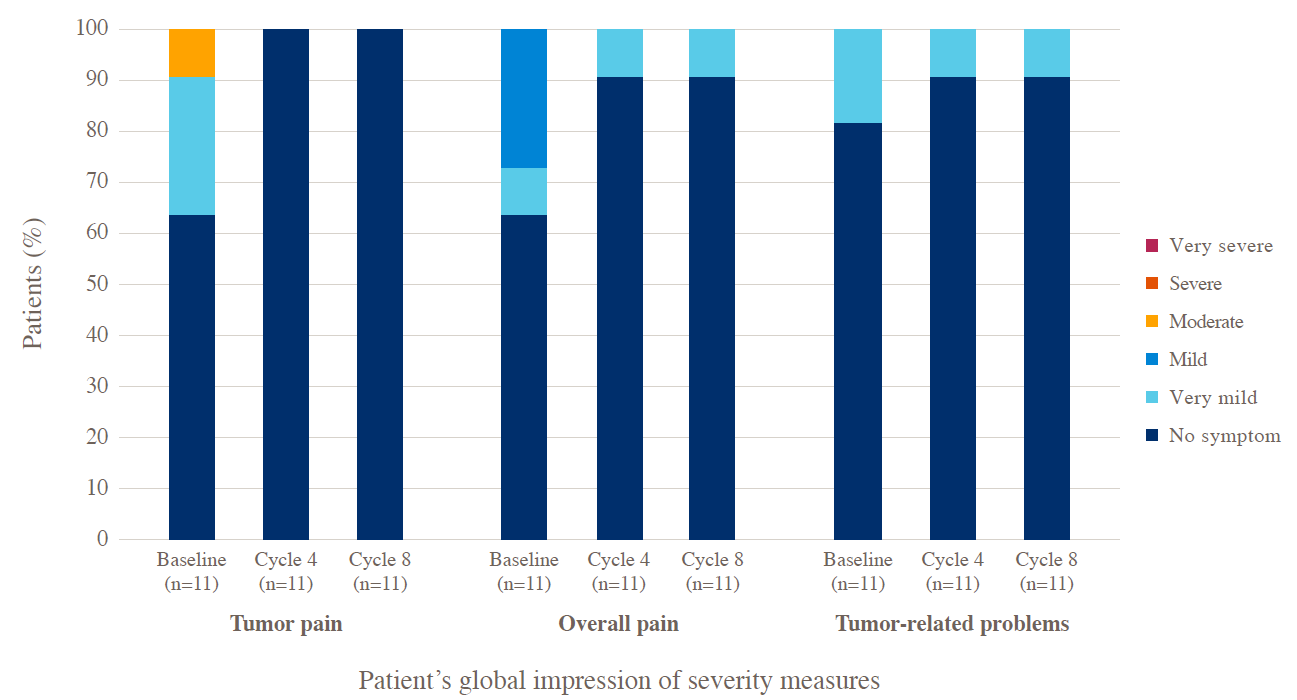
**

**C
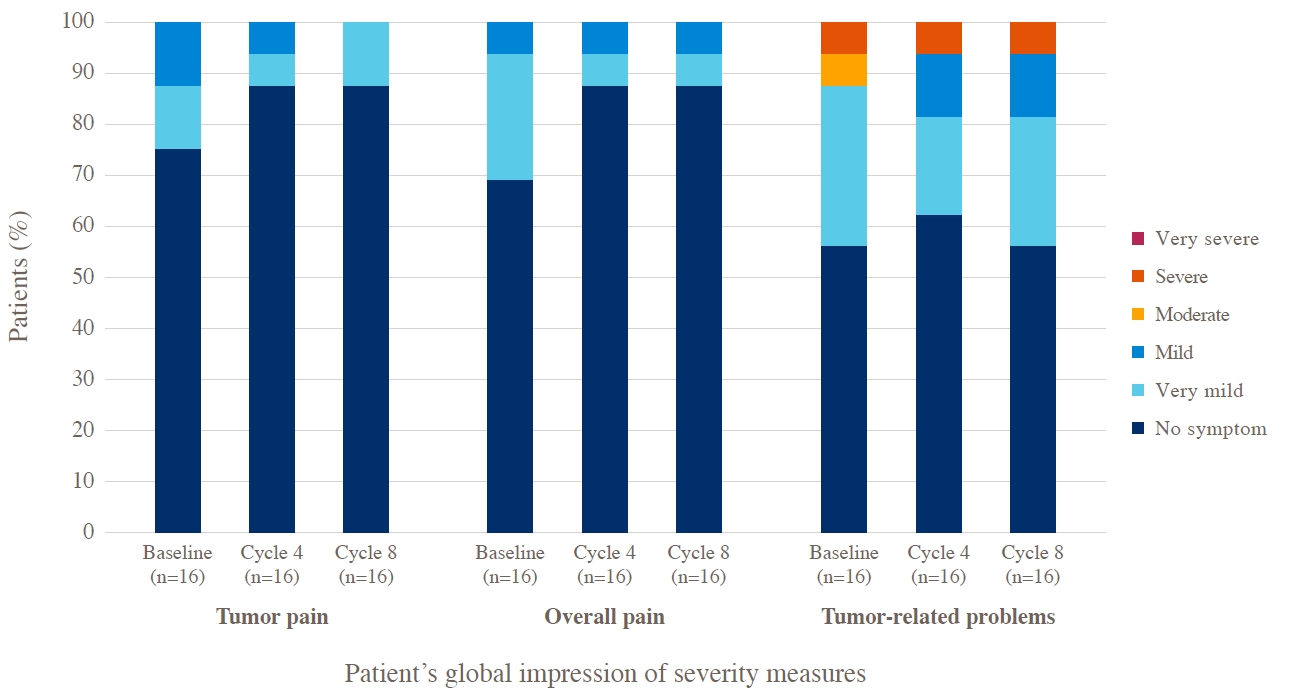
**

## **5 SUPPLEMENTARY MATERIALS REFERENCES**

1. Dombi E, Ardern-Holmes SL, Babovic-Vuksanovic D, et al. Recommendations for imaging tumor response in neurofibromatosis clinical trials. *Neurology*. Nov 19 2013;81(21 Suppl 1):S33-S40. doi:10.1212/01.wnl.0000435744.57038.af

2. Downie WW, Leatham PA, Rhind VM, Wright V, Branco JA, Anderson JA. Studies with pain rating scales. *Ann Rheum Dis*. Aug 1978;37(4):378-81. doi:10.1136/ard.37.4.378

3. Tomlinson D, von Baeyer CL, Stinson JN, Sung L. A systematic review of faces scales for the self-report of pain intensity in children. *Pediatrics*. Nov 2010;126(5):e1168-e1198. doi:10.1542/peds.2010-1609

4. Martin S, Nelson Schmitt S, Wolters PL, et al. Development and validation of the English Pain Interference Index and Pain Interference Index-Parent report. *Pain Med*. Feb 2015;16(2):367-73. doi:10.1111/pme.12620

5. Holmström L, Kemani MK, Kanstrup M, Wicksell RK. Evaluating the statistical properties of the Pain Interference Index in children and adolescents with chronic pain. *J Dev Behav Pediatr*. Jul-Aug 2015;36(6):450-4. doi:10.1097/dbp.0000000000000191

6. DeWalt DA, Gross HE, Gipson DS, et al. PROMIS(®) pediatric self-report scales distinguish subgroups of children within and across six common pediatric chronic health conditions. *Qual Life Res*. Sep 2015;24(9):2195-208. doi:10.1007/s11136-015-0953-3

7. Fayers P, Bottomley A. Quality of life research within the EORTC-the EORTC QLQ-C30. *Eur J Cancer*. Mar 2002;38(Suppl 4):S125-S133. doi:10.1016/s0959-8049(01)00448-8

8. Heaney A, Wilburn J, Rouse M, et al. The development of the PlexiQoL: a patient-reported outcome measure for adults with neurofibromatosis type 1-associated plexiform neurofibromas. *Mol Genet Genomic Med*. Dec 2020;8(12):e1530. doi:10.1002/mgg3.1530

9. Varni JW, Seid M, Kurtin PS. PedsQL 4.0: reliability and validity of the Pediatric Quality of Life Inventory version 4.0 generic core scales in healthy and patient populations. *Med Care*. Aug 2001;39(8):800-12. doi:10.1097/00005650-200108000-00006

10. AstraZeneca. Koselugo approved in China for paediatric patients with neurofibromatosis type 1 and plexiform neurofibromas. Accessed September 7, 2023. <https://www.astrazeneca.com/media-centre/press-releases/2023/koselugo-approved-in-china-for-paediatric-patients-with-neurofibromatosis-type-1-and-plexiform-neurofibromas.html>

11. AstraZeneca. Koselugo approved in Japan for paediatric patients with plexiform neurofibromas in neurofibromatosis type 1. Accessed September 7, 2023. <https://www.astrazeneca.com/media-centre/press-releases/2022/koselugo-approved-in-japan-for-paediatric-patients-with-plexiform-neurofibromas.html>

12. U.S. Food and Drug Administration. Selumetinib (Koselugo) Full Prescribing Information. Accessed September 7, 2023. <https://www.accessdata.fda.gov/drugsatfda_docs/label/2020/213756s000lbl.pdf>

13. Gross AM, Singh G, Akshintala S, et al. Association of plexiform neurofibroma volume changes and development of clinical morbidities in neurofibromatosis 1. *Neuro Oncol*. Nov 12 2018;20(12):1643-1651. doi:10.1093/neuonc/noy067

14. Baldo F, Magnolato A, Barbi E, Bruno I. Selumetinib side effects in children treated for plexiform neurofibromas: first case reports of peripheral edema and hair color change. *BMC Pediatr*. Feb 6 2021;21(1):67. doi:10.1186/s12887-021-02530-5

15. Wise JB, Cryer JE, Belasco JB, Jacobs I, Elden L. Management of head and neck plexiform neurofibromas in pediatric patients with neurofibromatosis type 1. *Arch Otolaryngol Head Neck Surg*. Aug 2005;131(8):712-8. doi:10.1001/archotol.131.8.712

16. Gross AM, Wolters PL, Dombi E, et al. Selumetinib in children with inoperable plexiform neurofibromas. *N Engl J Med*. Apr 9 2020;382(15):1430-1442. doi:10.1056/NEJMoa1912735

17. Neurofibromatosis. Conference statement. National Institutes of Health Consensus Development Conference. *Arch Neurol*. May 1988;45(5):575-8.
